# Comparative genomics of *Leishmania* (*Mundinia*)

**DOI:** 10.1186/s12864-019-6126-y

**Published:** 2019-10-11

**Authors:** Anzhelika Butenko, Alexei Y. Kostygov, Jovana Sádlová, Yuliya Kleschenko, Tomáš Bečvář, Lucie Podešvová, Diego H. Macedo, David Žihala, Julius Lukeš, Paul A. Bates, Petr Volf, Fred R. Opperdoes, Vyacheslav Yurchenko

**Affiliations:** 1 Life Science Research Centre, Faculty of Science, University of Ostrava, Ostrava, Czech Republic; 2 Biology Centre, Institute of Parasitology, Czech Academy of Sciences, České Budejovice (Budweis), Czech Republic; 3 Zoological Institute of the Russian Academy of Sciences, St Petersburg, Russia; 4 Department of Parasitology, Faculty of Science, Charles University, Prague, Czech Republic; 5 Martsinovsky Institute of Medical Parasitology, Tropical and Vector Borne Diseases, Sechenov University, Moscow, Russia; 6 Faculty of Sciences, University of South Bohemia, České Budejovice (Budweis), Czech Republic; 7 Division of Biomedical and Life Sciences, Faculty of Health and Medicine, Lancaster University, Lancaster, UK; 8 de Duve Institute, Université Catholique de Louvain, Brussels, Belgium

**Keywords:** Whole genome sequencing, *Leishmania* (*Mundinia*) *enriettii*, *L.* (*M*.) *macropodum*, *L.* (*M*.) *martiniquensis*

## Abstract

**Background:**

Trypanosomatids of the genus *Leishmania* are parasites of mammals or reptiles transmitted by bloodsucking dipterans. Many species of these flagellates cause important human diseases with clinical symptoms ranging from skin sores to life-threatening damage of visceral organs. The genus *Leishmania* contains four subgenera: *Leishmania*, *Sauroleishmania*, *Viannia*, and *Mundinia*. The last subgenus has been established recently and remains understudied, although *Mundinia* contains human-infecting species. In addition, it is interesting from the evolutionary viewpoint, representing the earliest branch within the genus and possibly with a different type of vector. Here we analyzed the genomes of *L*. (*M*.) *martiniquensis*, *L*. (*M*.) *enriettii* and *L.* (*M*.) *macropodum* to better understand the biology and evolution of these parasites.

**Results:**

All three genomes analyzed were approximately of the same size (~ 30 Mb) and similar to that of *L.* (*Sauroleishmania*) *tarentolae*, but smaller than those of the members of subgenera *Leishmania* and *Viannia*, or the genus *Endotrypanum* (~ 32 Mb). This difference was explained by domination of gene losses over gains and contractions over expansions at the *Mundinia* node, although only a few of these genes could be identified. The analysis predicts significant changes in the *Mundinia* cell surface architecture, with the most important ones relating to losses of LPG-modifying side chain galactosyltransferases and arabinosyltransferases, as well as β-amastins. Among other important changes were gene family contractions for the oxygen-sensing adenylate cyclases and FYVE zinc finger-containing proteins.

**Conclusions:**

We suggest that adaptation of *Mundinia* to different vectors and hosts has led to alternative host-parasite relationships and, thereby, made some proteins redundant. Thus, the evolution of genomes in the genus *Leishmania* and, in particular, in the subgenus *Mundinia* was mainly shaped by host (or vector) switches.

## Background

Obligate flagellate parasites of the family Trypanosomatidae infect insects, leeches, vertebrates, and plants [1–3]. They have one (monoxenous species) or two hosts (dixenous species) in their life cycle [4–6]. Dixenous representatives belong to the genera *Endotrypanum*, *Leishmania*, *Paraleishmania*, *Phytomonas*, and *Trypanosoma* and some of them are of medical and/or economic importance [7–9]. It is generally accepted that all dixenous trypanosomatids have originated from their monoxenous kin [10]. Supporting this, in the current taxonomical system, the dixenous genera *Endotrypanum*, *Leishmania*, *Paraleishmania* are united with the monoxenous genera *Borovskyia*, *Crithidia*, *Leptomonas*, *Lotmaria*, *Novymonas*, and *Zelonia* into the subfamily Leishmaniinae [11, 12], while the dixenous genus *Phytomonas* is included into subfamily Phytomonadinae along with the monoxenous genera *Herpetomonas* and *Lafontella* [13].

Parasites of the genus *Leishmania* infect mammals or reptiles and cause various diseases named leishmaniases. For humans, this translates into over 350 million people being at risk of infection primarily in the tropical and subtropical regions [14]. These parasites are transmitted by bloodsucking phlebotomine sand flies (Psychodidae) or, possibly, biting midges (Ceratopogonidae) [15, 16] and manifest the infection by a range of clinical symptoms from innocuous skin lesions to fatal visceral organ failures [7].

Currently, the following four subgenera are recognized within the genus *Leishmania*. These are *Leishmania* (*Leishmania*), *L.* (*Mundinia*), *L.* (*Sauroleishmania*), and *L.* (*Viannia*) [17]. They are not only well-defined phylogenetically, but can also be delineated by host specificity or clinical picture. The most enigmatic of them is *Mundinia* [18], the last established subgenus [17], which, as of now, contains only four described species: *L. enriettii*, *L. macropodum*, *L. martiniquensis*, and *L. orientalis* [19–22]. In addition, there are isolates from Ghana, likely representing a separate species, which is phylogenetically close to *L. orientalis* [20].

*Leishmania* (*Mundinia*) spp. are of special interest for, at least, four main reasons. Firstly, in this group, human pathogens – *L*. (*M*.) *orientalis*, *L*. (*M*.) *martiniquensis* and parasites from Ghana – are intermingled with species non-pathogenic to humans, namely *L*. (*M*.) *enriettii* and *L.* (*M*.) *macropodum* [20, 23]. *Leishmania* (*M*.) *enriettii* infects guinea pigs in South America [24, 25], while *L.* (*M*.) *macropodum* was found in Australian macropods [26, 27]. In addition, parasites apparently belonging to *L. martiniquensis* have been also recorded in cows and horses [28–30]. Secondly, a significant portion of human patients infected with *Leishmania* (*Mundinia*) are immunocompromised [31–33], indicating that these parasites may actively explore new developmental niches [10, 34]. A similar situation has been documented in some thermo-tolerant monoxenous trypanosomatids [35–37]. Thirdly, *Mundinia* spp. may be transmitted primarily not by phlebotomine sand flies of the genera *Phlebotomus* and *Lutzomyia* as for other leishmaniae, but by biting midges or other genera of sand flies, although more work is needed to confirm this with certainty [15, 38]. Fourthly, and finally, in all phylogenetic reconstructions, *L.* (*Mundinia*) represents the earliest branch within the genus *Leishmania*, suggesting its ancient origin prior to the breakup of Gondwana [2, 39].

For all these reasons, members of the subgenus *Mundinia* qualify as crucial for comparative genomic analyses, as they may shed light on the evolution of *Leishmania* and its pathogenicity for humans. Similar analyses have been done and reported for *L.* (*Sauroleishmania*) [40, 41], *L.* (*Viannia*) [42–45], *L.* (*Leishmania*) [46, 47], leaving *Mundinia* understudied in this respect.

In this work, we sequenced and analyzed genomes of three *Leishmania* (*Mundinia*) species, which represent the major clades of the subgenus: *L.* (*M.*) *enriettii* MCAV/BR/1945/LV90 originating from southern Brazil, *L.* (*M*.) *macropodum* MMAC/AU/2004/AM-2004 originating from northern Australia, and *L.* (*M*.) *martiniquensis* MHOM/MQ/1992/MAR1 originating from the Caribbean island of Martinique. The genomic sequence of *L.* (*M.*) *enriettii* MCAV/BR/1945/LV90 complemented a previously obtained one, which belongs to a different isolate of the same species (MCAV/BR/1995/CUR3) and is available from the TriTryp database.

## Methods

### Origin of isolates, cultivation, amplification, sequencing and species verification

Promastigotes were cultured in the M199 medium (Sigma−Aldrich, St. Louis, MO, United States) containing 10% heat-inactivated fetal bovine calf serum (FBS; Thermo Fisher Scientific, Waltham, MA, United States), supplemented with 1% Basal Medium Eagle vitamins (Sigma−Aldrich), 2% sterile urine and 250 μg/ml of amikacin (Bristol-Myers Squibb, New York, NY, United States).

Total genomic DNA was isolated from 10 ml of trypanosomatid cultures with the DNeasy Blood & Tissue Kit (Qiagen, Hilden, Germany) according to the manufacturer’s instructions. 18S rRNA gene was amplified using primers S762 and S763 [48], following the previously described protocol [13]. These PCR fragments were sequenced directly at Macrogen Europe (Amsterdam, Netherlands) as described previously [49]. The identity of species under study was confirmed by BLAST analysis [50].

### Whole-genome and whole-transcriptome sequencing and analysis

The genomes and whole transcriptomes of *Leishmania* (*Mundinia*) isolates were sequenced as described previously [35, 51, 52] using the Illumina HiSeq and NovaSeq technologies with TruSeq adapters for the libraries preparation, respectively, at Macrogen Inc. (Seoul, South Korea). 43 and 47 million 100 nt paired-end raw reads on average were produced for genomes and transcriptomes, respectively (see statistics below). The genome completeness and annotation quality were assessed using BUSCO software [53]. The raw reads were trimmed with Trimmomatic v. 0.32 [54] with the following settings: ILLUMINACLIP:TruSeq3-PE-2.fa:2:20:10 LEADING:3 TRAILING:3 SLIDINGWINDOW:4:15 MINLEN:75, quality-checked with FASTQC program v.0.11.5, and then assembled de novo with the Spades Genome assembler v. 3.10.1 with the default settings and automatic k-mer selection (k-mers of 21, 33 and 55 were used) [55]. The Trinity assembler v. 2.4.0 [56] was used to reconstruct the transcriptomes de novo with the minimal contig length of 150. Resulting genome assemblies were investigated for potential contamination using the BlobTools software implementing Bowtie2 [57] for genome read mapping and Hisat2 for transcriptome read mapping [58], both with the default settings. Only those read pairs were used where at least one read was present in some contig with the transcriptome read coverage higher than 10 or in a contig with *Leishmania*, *Leptomonas*, or *Trypanosoma* term in first 100 best Diamond hits. Other read pairs were filtered out (Additional file 1: Figure S1, Additional file 2: Figure S2, Additional file 3: Figure S3, Additional file 4: Figure S4, Additional file 5: Figure S5, Additional file 6: Figure S6). Resulting assemblies (CovPlots, Additional file 7: Figure S7, Additional file 8: Figure S8, Additional file 9: Figure S9) were further inspected and curated manually. Parameters of the genome assemblies were estimated using QUAST v. 4.5 [59]. Raw reads were submitted to NCBI SRA under accession numbers SRX5006814, SRX5006815, and SRX5006816 (Bioproject: PRJNA505413) for *L*. (*M*.) *enriettii* MCAV/BR/1945/LV90, *L*. (*M*.) *macropodum* MMAC/AU/2004/AM-2004, and *L*. (*M*.) *martiniquensis* MHOM/MQ/1992/MAR1, respectively.

Genome annotation was performed with the Companion software [60] using transcriptome evidence, *Leishmania major* as a reference organism, and pseudochromosome contiguation with default settings. Transcriptome evidence was generated with the Cufflinks, mapping was performed with the Hisat2 with --dta-cufflinks parameter [58].

### Synteny analysis

Synteny analysis was performed using SyMAP v. 4.2 [61] with the following settings: minimum size of sequence to load, 500 bp; minimum number of anchors required to define a synteny block, 7; synteny blocks were merged in case of overlaps, and only the larger block was kept if two synteny blocks overlapped on a chromosome. In case of *Leishmania* (*Mundinia*) genomes sequenced in this study, pseudochromosome level assembly built using Companion software with *L. major* Friedlin genome as a reference was used for the analysis instead of scaffolds in order to reduce computational time.

### Genome coverage analysis and ploidy estimation

Per-base read coverage was calculated for fifty longest scaffolds and all pseudochromosome level sequences using BEDTools v. 2.26.0 genomecov tool [62] on the read mappings generated with Bowtie2 as described above. Mean genome and scaffold/pseudochromosme coverage was calculated using a custom Python script. Ploidy was estimated based on relative coverage values: mean coverage for each of the fifty longest scaffolds and all psedochoromosome level sequences was divided by mean genome coverage and ploidy was inferred under the assumption that the majority of chromosomes are diploid. Coverage plots for 50 longest scaffolds were generated using weeSAM tool v. 1.5 (http://bioinformatics.cvr.ac.uk/blog/weesam-version-1-5/).

### Variant calling

Prior to variant calling, duplicates removal and local re-alignment were performed on the respective read mappings using GATK v. 4.1.2.0 MarkDuplicates and IndelRealigner tools with the following parameter differing from the default: --REMOVE_DUPLICATES = true [63]. Variant calling was performed using Platypus v. 0.1.5 [64] with the default settings and only SNPs were considered in further analyses.

### Inference of protein orthologous groups and phylogenomic analyses

Analysis of protein orthologous groups was performed on a dataset containing 41 trypanosomatid species (including four representatives of the subgenus *Mundinia,* Additional file 16: Table S1) and a eubodonid *Bodo saltans* as an outgroup, using OrthoFinder v. 1.1.8 with the default settings [65]. Out of a total 551 OGs containing only one protein for each species, 92 were selected for the phylogenomic inference according to the following criteria: i) average percent identity within the group ≥60%; ii) maximum percentage of gaps per sequence in the alignment before trimming – 40%; iii) maximum percentage of gaps per sequence in the alignment after trimming – 10%. The amino acid sequences of each gene were aligned using Muscle v. 3.8.31 [66]. The average percent identity within each OG was calculated using the *alistat* script from the HMMER package v.3.1 [67]. The alignments were trimmed using trimAl v. 1.4.rev22 with the “-strict” option [68]. The resulting concatenated alignment contained 32,460 columns. The maximum likelihood tree was inferred in IQ-TREE v. 1.6.3 with the JTT + F + I + G4 model and 1000 bootstrap replicates [69, 70]. For the construction of the Bayesian tree PhyloBayes-MPI 1.7b was run for over 9000 iterations under the GTR-CAT model with four discrete gamma categories [71]. Every second tree was sampled and first 25% of them were discarded as “burn-in”. The final tree was visualized using FigTree v.1.4.3 (http://tree.bio.ed.ac.uk/software/figtree/). Gains/losses and expansions/contractions of protein families were analyzed using the COUNT software with Dollo’s and Wagner’s (gain penalty set to 3) parsimony algorithms, respectively [72]. For gene ontology (GO) annotation of gene families gained/lost/expanded/contracted at certain nodes Blast2GO Basic software [73] was used with the maximum number of BLAST hits set to 10 and other settings left as default. Assignment of KEGG IDs to the proteins of interest was performed via BlastKOALA server with a target database of eukaryotes and prokaryotes at the family and genus levels, respectively [74]. The analysis of OGs shared among *Leishmania* was performed using UpSetR package [75].

### Analysis of amastin repertoire

Amastin sequences of *L. major* Friedlin, *Trypanosoma brucei* TREU927, and *Trypanosoma cruzi* CL Brener Esmeraldo were downloaded from the TriTrypDB release 41 and used as queries in BLAST search with an E-value threshold of 10^− 20^ against a database of annotated proteins of *Crithidia fasciculata*, *Endotrypanum monterogeii*, *Leishmania braziliensis* MHOM/BR/75/M2904, *Leishmania* (*Mundinia*) spp., *Leptomonas pyrrhocoris* H10, and *Trypanosoma grayi* ANR4. The resulting sequences were aligned using Muscle v.3.8.31 with the default parameters [66]. *P*-distances were calculated using MEGA 7 software [76], and the hits with *p*-distance to the α-amastin of *T. brucei* (Additional file 17: Table S2) exceeding 0.9 and query coverage < 50% were excluded from further analyses. The resulting alignment was trimmed using TrimAl v.1.4.rev22 with the ‘-gappyout’ option [68]. Maximum-likelihood phylogenetic tree was inferred on the final dataset containing 384 sequences and 436 characters using IQ-TREE v.1.5.3 with the VT + F + G4 model and 1000 bootstrap replicates [69, 70].

### Analysis of side chain galactosyltransferases

The identification of the side chain galactosyltransferases (SCGs) was performed as described previously [77]. Proteins with *p*-distances to SCGs of *L. major* exceeding 0.8 were excluded from further analysis (Additional file 18: Table S3 and Additional file 19: Table S4). Phylogenetic reconstruction was performed using IQ-TREE v.1.5.3 with 1000 bootstrap replicates and VT + F + I + G4 and JTT + F + G4 models for the SCGs and side chain arabinosyltransferases (SCAs), respectively.

### Analyses of other proteins within OGs gained/lost at certain nodes

For the identification of putative phosphatydylinositol glycan class Y proteins (PIG-Y), we have performed sensitive homology searches using the HMMER package v.3.1 [67] and a model build using aligned sequences of trypanosomatid annotated as PIG-Y from the TriTrypDB release 41 [78]. Phylogenetic analysis of PIG-Y was performed similarly to amastins, with the JTT + I + G4 model as best-fitting and excluding sequences with *p*-distances to the reference set higher than 0.8 (Additional file 20: Table S5). The analysis of ferrochelatase sequences was performed similarly (Additional file 21: Table S6), with the JTT + I + G4 phylogenetic model.

## Results

### Assembly and annotation of three *Leishmania* (*Mundinia*) genomes

The three sequenced genomes were assembled and annotated, yielding total lengths of 29.95, 29.59, and 29.83 Mbp for *L*. (*M*.) *martiniquensis* MHOM/MQ/1992/MAR1, *L*. (*M*.) *macropodum* MMAC/AU/2004/AM-2004, and *L*. (*M*.) *enriettii* MCAV/BR/1945/LV90, respectively for the scaffolds longer than 500 bp (Additional file 22: Table S7). The N50 values and largest scaffold sizes varied from 24.17 to 33.45 kbp, and from 181 to 225 kbp for *L*. (*M*.) *enriettii* and *L*. (*M*.) *martiniquensis*, respectively. Genomic reads coverage analysis (Additional file 10: Figure S10) indicates that coverage is fairly uniform across *Mundinia* genome assemblies, with the regions of coverage close to median values (exceeding 40x but lower than 150x) combined together accounting for ~ 91, 89 and 80% of genome assembly length for *L*. (*M*.) *martiniquensis*, *L*. (*M*.) *macropodum*, and *L*. (*M*.) *enriettii*, respectively. The results of variant calling suggest that the genome of *L*. (*M*.) *enriettii* carrying 12,379 SNPs is characterized by higher variation levels than those of *L*. (*M*.) *martiniquensis* and *L*. (*M*.) *macropodum* with 1765 and 4834 identified SNPs, respectively (Additional file 22: Table S7). The number of homozygous SNPs identified in *L*. (*M*.) *martiniquensis*, *L*. (*M*.) *macropodum*, and *L*. (*M*.) *enriettii* genome assemblies were as low as 64, 67 and 121, respectively, suggesting minimal number of misassembly events (Additional file 22: Table S7).

Expectedly, the results of ploidy analysis suggest that *Leishmania* (*Mundinia*) spp. demonstrate variable degree of aneuploidy (Additional file 23: Table S8). In *L*. (*M*.) *martiniquensis* all pseudochromosome level sequences appear to be diploid, except for chromosome 31. The genome of *L*. (*M*.) *enriettii* displays the highest level of aneuploidy among the analyzed species, with nine chromosomes of variable ploidy levels (Additional file 23: Table S8).

**Fig. 1:**
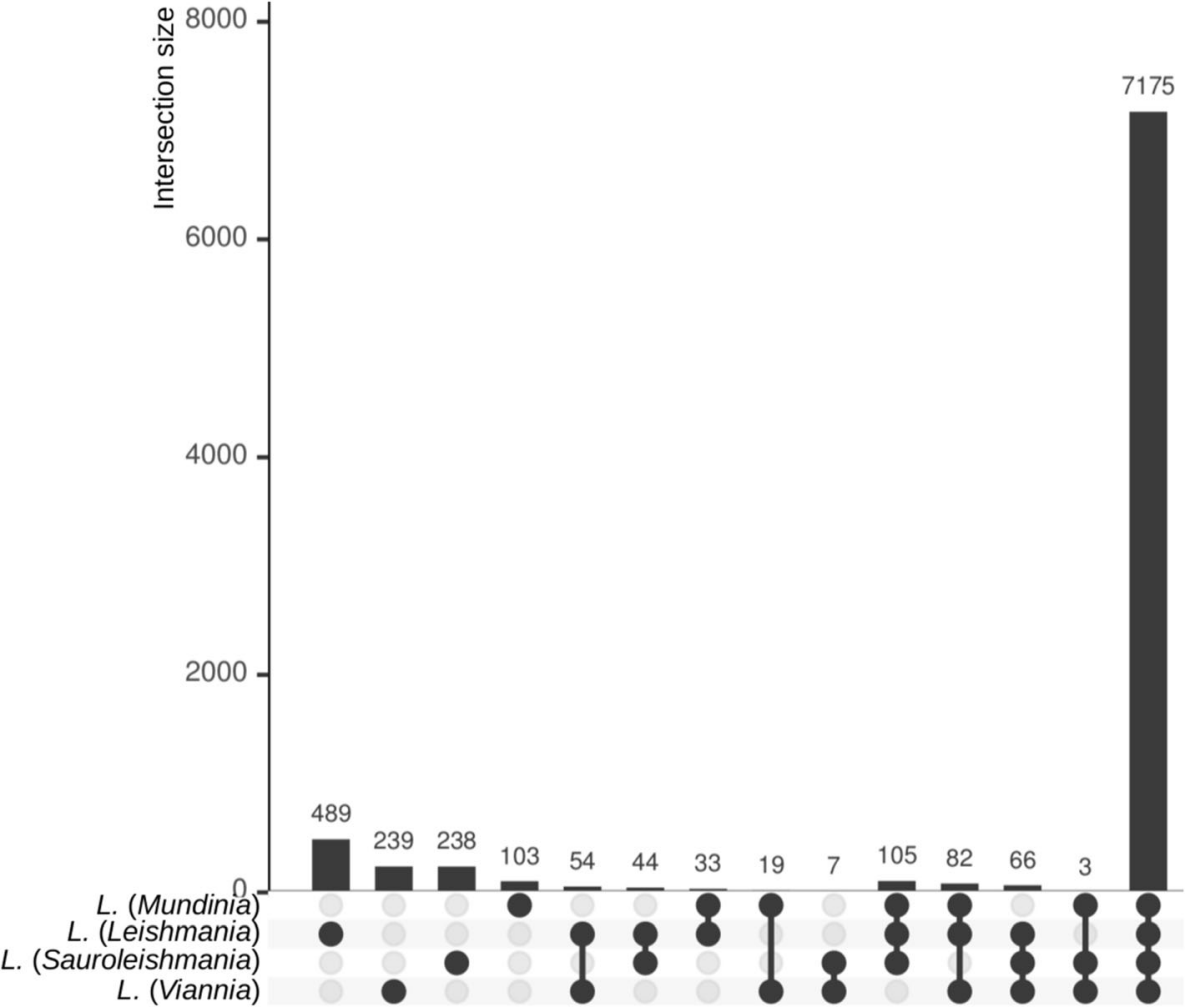
The phyletic patterns for OGs identified in four subgenera of the genus *Leishmania*: *L*. (*Leishmania*), *L*. (*Viannia*), *L*. (*Mundinia*), and *L*. (*Sauroleishmania*). An UpSetR plot shows the numbers of orthologous groups uniquely shared among four subgenera of *Leishmania*. Intersection size (the number of shared OGs) is plotted on Y axis; dataset intersection options are indicated on the X axis with black circles

All the analyzed genomes are predicted to encode around 8000 genes and had complete BUSCOs percentage of around 72% (Additional file 22: Table S7). For comparison, the previously sequenced genome of another isolate of *L*. (*M*.) *enriettii* – MCAV/BR/1995/CUR3 (LEM3045) – has similar, albeit slightly larger (partially due to a ~ 60-fold higher gap content), size of 30.9 Mbp (29.2 Mbp in 36 scaffolds) and was predicted to encode 8831 genes. *Mundinia* genomes obtained in this study show high degree of synteny to publicly available ones and the assembly for *L. major* Friedlin (Additional file 11: Figure S11). From 93 to 98% of genes identified in the assemblies obtained in this study are located within synteny blocks in various intra- and interspecies comparisons (Additional file 11: Figure S11, panel B). The absence of collapsed repeats and highly similar genes in the obtained assemblies is supported by the absence of regions of double coverage (i.e., regions covered by two or more synteny blocks) as compared to publicly available genomes (Additional file 11: Figure S11, panel B). Annotated proteins of all representatives of the genus *Leishmania* within our dataset cluster into 8657 OGs. Most of these groups (83%, 7175 OGs) are shared among all four subgenera (Fig. 1). *Mundinia* spp. appear to possess the lowest number of the subgenus-specific OGs (~ 100), while the representatives of *L*. (*Leishmania*) have ~ 500 such groups.

### Phylogenomic analysis

**Fig. 2:**
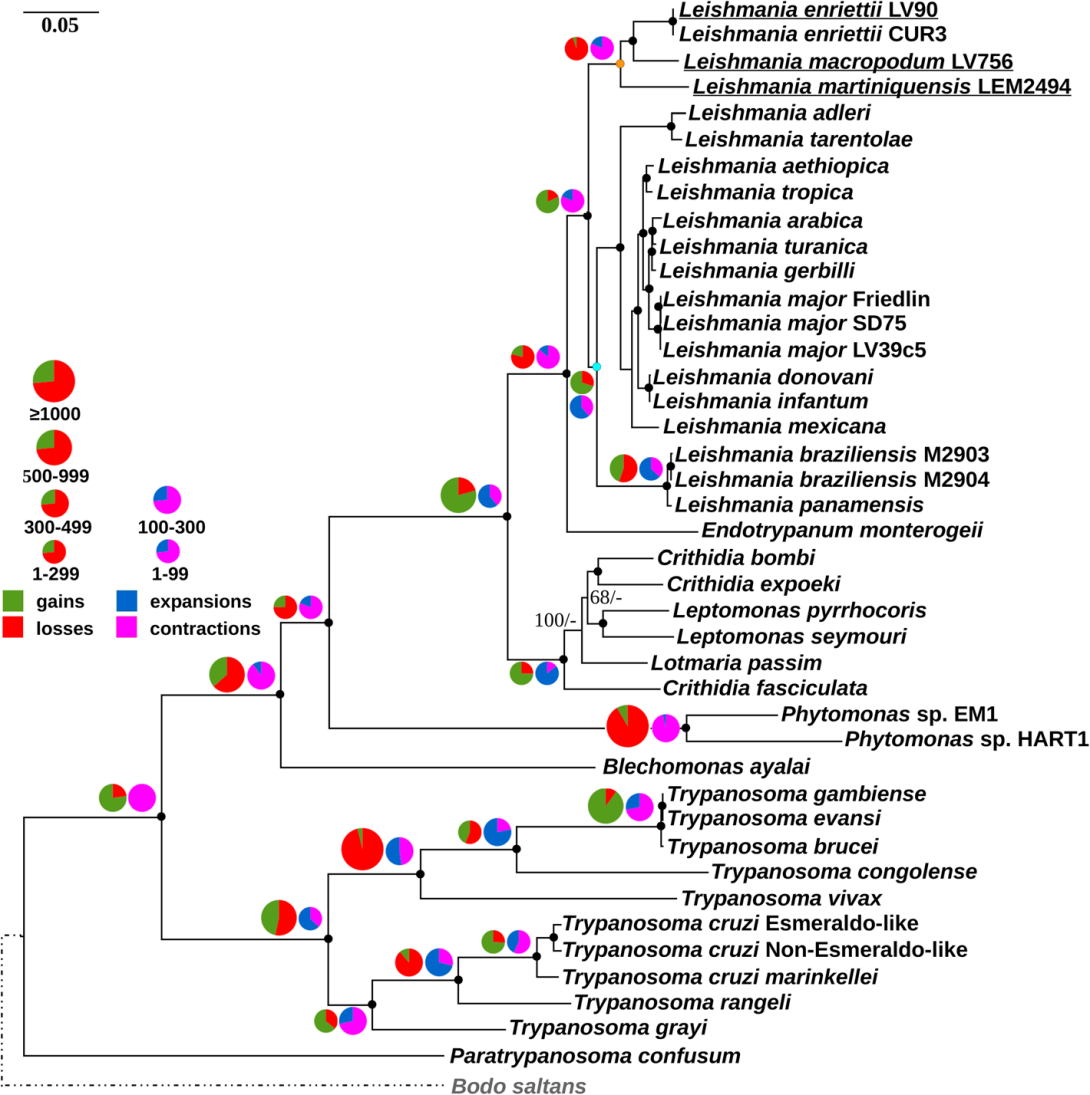
The phylogenetic tree of trypanosomatids and *Bodo saltans* based on the alignment of 92 conserved proteins. Only bootstrap support values lower than 100% and posterior probabilities lower than 1 are shown. The scale bar represents 0.05 substitutions per site. Pie charts depict relative proportions of OGs gains/losses and expansions/contractions in green/red and blue/magenta colors, respectively. The area of the pie charts is proportional to a total number of OGs gained/lost or expanded/contracted at a certain node. The nodes corresponding to the subgenus *Mundinia* and to the all other *Leishmania* are highlighted in orange and cyan, respectively

The Maximum Likelihood and Bayesian trees inferred using the matrix of 92 single-copy OGs displayed identical topologies with almost all branches having maximal bootstrap percentage and posterior probabilities (except for two modestly resolved branches of monoxenous Leishmaniinae: *Lotmaria passim* and intermingled species of the *Leptomonas* – *Crithidia* clade). Our results confirmed the phylogenetic position of *Mundinia* as the earliest branch within the genus *Leishmania* (Fig. 2), which has been inferred in previous studies [2, 39]. It is also in agreement with the recently published phylogenetic trees of *Mundinia* spp., which were reconstructed using several single phylogenetic markers [20, 23].

### Gene gains and losses at the *Leishmania* (*Mundinia*) node

The *Leishmania* (*Mundinia*) node was heavily dominated by gene losses. There were 13 gained and 234 lost OGs at this node (Fig. 2, Additional file 24: Table S9). All 13 gained and 148 lost OGs contained genes encoding hypothetical proteins. In contrast, the node uniting the three remaining subgenera was dominated by gene gains with 79 gained (71 OGs contained genes encoding hypothetical proteins) and 34 lost (22 OGs contained genes encoding hypothetical proteins) (Fig. 2, Additional file 25: Table S10).

The annotations for sequences within OGs lost at the *L*. (*Mundinia*) node indicate changes in the surface architecture of the parasites of this subgenus, exemplified by the losses of putative amastins, glycosylphosphatidylinositol (GPI) anchor biosynthesis and turnover proteins. Amastins are a large family of surface glycoproteins, highly expressed in the amastigote stage of several trypanosomatids, such as *T. cruzi* and *Leishmania* spp. [79]. They are essential for establishing infection in macrophages [80, 81] and, therefore, are significantly reduced in lizard-parasitizing *L. tarentolae*, which cannot efficiently replicate in this type of cells and rarely forms amastigotes [41].

The results of our gene content evolution analyses suggest that three OGs containing putative amastins were lost at the *L*. (*Mundinia*) node (Additional file 24: Table S9). According to the phylogenetic analysis (Additional file 12: Figure S12), two of those OGs – OG0008773 and OG0009479 (Additional file 24: Table S9) – contain putative β-amastin-like proteins, homologues of which were lost in all analyzed *Leishmania* spp. except for *L. major* and *L. braziliensis*, respectively. OG0009537 incorporates γ-amastin-related proteins, identified in the genomes of the monoxenous Leishmaniinae, but lost in all *L.* (*Leishmania*) spp. [82]. Overall, 33, 19 and 23 amastin-like sequences were identified in *L*. (*M*.) *martiniquensis*, *L*. (*M*.) *macropodum*, and *L*. (*M*.) *enriettii*, respectively. *L*. (*Mundinia*) genomes encode representatives of all four amastin subfamilies, including *Leishmania*-specific δ-amastins.

The amastin polypeptides are linked to the parasite’s outer membrane via a GPI anchor [83, 84]. Two enzymes involved in GPI-anchor synthesis and GPI-anchored protein turnover, phosphatidylinositol N-acetylglucosaminyltransferase (subunit Y) and glycosylphosphatidylinositol phospholipase-C (GPI-PLC), respectively, also appear to be lost at the *L*. (*Mundinia*) node. However, a careful inspection of the results has shown that GPI-PLC is absent not only from *Mundinia*, but also from other subgenera of *Leishmania*, as well as from *Endotrypanum*. The only exception is *L. panamensis* with a partial sequence of unknown function returning a short hit to the GPI-PLC. This hit resulted in erroneous inference of the putative GPI-PLC presence at the *L.* (*Leishmania*) node by the Dollo’s parsimony algorithm. Putative GPI-PLC have been identified in all species within our dataset, except for dixenous Leishmaniinae, *C. expoeki*, and *Phytomonas* spp. In trypanosomatids, phosphatidylinositol N-acetyl-glucosaminyl-transferase, the enzyme catalyzing the first step of GPI biosynthesis, is composed of seven proteins: phosphatydyl-inositol glycan class A (PIG-A), PIG-C, PIG-H, PIG-Q, PIG-P, PIG-Y, and dolichyl-phosphate mannosyl-transferase polypeptide 2 (DPM2) [85]. All these proteins were identified in *L*. (*Mundinia*), with the exception of DMP2 and PIG-Y being absent from the genome of *L*. (*M*.) *macropodum*. The analysis of orthologous groups revealed that PIG-Y sequences fall into two different OGs, one of which appears to be absent in *L*. (*Mundinia*). More sensitive HMM-based searches led to the identification of PIG-Y proteins in several other trypanosomatids. The phylogenetic analysis confirmed the presence of two separate groups of PIG-Y sequences, only one of which contains *L*. (*Mundinia*) subunits (Additional file 13: Figure S13). Most of the *L.* (*Leishmania*) sequences fall into the latter group, while the representatives of the other clade appear to be in the process of pseudogenization in *L.* (*Leishmania*), as suggested by the presence of the identifiable pseudogenes in *L. major* and *L*. *tarentolae*.

We have also analyzed the repertoire of side chain galactosyltransferases (SCGs) and side chain arabinosyltransferases (SCAs), performing chemical modifications of the GPI-anchored lipophosphoglycan (LPG) on the cell surface of the Leishmaniinae [77, 86, 87], with the potential effect on host-parasite interactions [88–90]. The genome of *L*. (*M*.) *martiniquensis* encodes five SCGs, while those of *L*. (*M*.) *macropodum* and *L*. (*M*.) *enriettii*, sequenced in this study, contain four putative members of SCG/L/R family (Additional file 14: Figure S14). Thus, in *L.* (*Mundinia*) the number of SCG-encoding genes is substantially lower than in *L. major*, *L. braziliensis* and *L. infantum*, carrying 14, 17 and 12 genes, respectively. *L.* (*Mundinia*) SCG proteins cluster with those of *L. braziliensis*, and together they form a sister clade to the SCGs of *L. major* and *L. infantum*. In addition, *L.* (*Mundinia*) spp. contain sequences related to the SCGR1–6, while putative SCGL-encoding genes were not identified, similarly to the situation observed in *L. braziliensis* [91, 92]. Overall, the SCG/L/R repertoire in *L.* (*Mundinia*) is most similar to the one in *L. braziliensis*, with the exception of the SCG expansion in *L. braziliensis*, which is not documented in *L.* (*Mundinia*). In addition, *L.* (*Mundinia*) spp. possess SCA and SCA-like sequences, which are absent in *L. braziliensis* (Additional file 14. Figure S14).

A few genes encoding metabolic proteins appear to be lost in *L*. (*Mundinia*). An important enzyme of folate metabolism is methylene-tetrahydrofolate reductase (MTFR), which converts 5-methyltetrahydrofolate into 5,10-methylene-tetrahydrofolate and is required for the formation of activated C1 units used in the synthesis of both thymidylate by thymidylate synthase/dihydrofolate reductase and of methionine from cysteine by methionine synthase [93, 94]. MTFR is present in *Bodo saltans*, *Paratrypanosoma confusum*, *Blechomonas alayai*, and all Leishmaniinae with the sole exception of *L.* (*Mundinia*). In addition to this, it is also absent from trypanosomes and *Phytomonas*. However, the absence of MTFR does not imply auxotrophy for methionine, since all trypanosomatids seem to be able to synthesize this amino acid by an alternative route using homocysteine S-methyltransferase [95].

Following the observation that ferrochelatase (FeCH), the terminal enzyme in the heme biosynthetic pathway catalyzing the insertion of iron into protoporphyrin IX [96], was lost in *Leishmania* (Additional file 25. Table S10), we have checked the presence of other enzymes of this pathway. Some trypanosomatids (*Trypanosoma* and *Kentomonas*), have lost the heme biosynthetic pathway completely, while others retained genes encoding the last three enzymes (Leishmaniinae, *Angomonas* and *Strigomonas*), or only ferrochelatase (*Phytomonas* and *Herpetomonas*) [97–101]. Protoporphyrin IX, a substrate of FeCH, is synthesized by a subsequent action of coproporphyrinogen oxidase and protoporphyrinogen oxidase [102]. Both enzymes were readily identifiable in the genomes of *L.* (*Mundinia*) spp., except for *L*. (*M*.) *macropodum*. Sequences of FeCH clustered in two separate OGs, only one of which incorporates the proteins of all three *L.* (*Mundinia*) spp. (Additional file 15: Figure S15). The other OG contains only the sequences of *B. ayalai*, *E. monterogeiii*, *Phytomonas* spp., and monoxenous representatives of the subfamily Leishmaniinae. The phylogenetic analysis of FeCH (Additional file 15: Figure S15) suggests the presence of two divergent sequences encoding this protein in the genomes of trypanosomatids, which is in agreement with the results of previous studies concluding that there might have been two different FeCH LGT events from bacteria to kinetoplastids [99]. Indeed, the FeCH sequences of *C. fasciculata*, falling into two different clades, exhibit only ~ 22% identity, giving best BLAST hits outside the Euglenozoa to the γ-proteobacterial sequences.

Kinetoplastids lack the capacity of de novo lysine biosynthesis. However, *B. saltans*, *Leptomonas* and *Crithidia* spp. use the enzyme diaminopimelate epimerase (DAP) to convert diaminopimelate, an amino acid present in the cell walls of gram-negative bacteria, into lysine [97]. In all other trypanosomatids, including *L*. (*Mundinia*), DAP has been lost. The loss of genes encoding this enzyme suggests that most of the trypanosomatids have lost their dependency on bacterial diaminopimelate and, thus, are lysine auxotrophs. Interestingly, the genomes of most *L*. (*Leishmania*) spp. still possess easily identifiable diaminopimelate epimerase pseudogenes, while no remnants of DAP-encoding genes could be found in other trypanosomatid genomes. This suggests that these genes could have been acquired by the common ancestor of all *Leishmaniinae* and then independently lost in different lineages of its dixenous descendants.

### Gene family expansions and contractions at the *Leishmania* (*Mundinia*) node

In *L.* (*Mundinia*), 9 gene families were expanded (3 genes encoding hypothetical proteins) and 40 contracted (7 genes encoding hypothetical proteins) (Fig. 2; Additional file 26: Table S11), while in other subgenera, 11 gene families were expanded (4 genes encoding hypothetical proteins) and 7 contracted (3 genes encoding hypothetical proteins) (Fig. 2; Additional file 27: Table S12). The degree of gene family expansion/contraction is rather moderate, with the family size changes involving from 1 to 5 gene copies (Additional file 26: Table S11, Additional file 27: Table S12).

Oxygen-sensing adenylate cyclases (OG0000628) govern O_2_-dependent cAMP signaling via protein kinase A, and, consequently, cell survival and proliferation of *Leishmania* promastigotes under low concentration of oxygen [103]. Contraction of this gene family in *L.* (*Mundinia*) suggests that these parasites either rely on different mechanisms to deal with hypoxia or are under different environmental cues during development in their vectors.

Another interesting example is a contracted gene family encoding FYVE zinc finger-containing proteins (OG0001095). In eukaryotes, the FYVE domain is responsible for the recruitment of proteins to different organelles such as multivesicular bodies, endosomes, or phagosomes [104]. Membrane recruitment is mediated by the binding of the FYVE domain to membrane-embedded phosphatidylinositol-3-phosphate [105]. Why this gene family is contracted in *L.* (*Mundinia*) remains to be investigated further.

## Discussion

The genomes of the three species of *Leishmania* (*Mundinia*) analyzed here are similar in size to that of *L.* (*Sauroleishmania*) *tarentolae* (~ 30 Mb), but smaller than those of the representatives of the subgenera *L.* (*Leishmania*) and *L.* (*Viannia*), as well as the genus *Endotrypanum* (~ 32 Mb). This correlates not only with the intuitively understandable domination of gene losses over gains and contractions over expansions, but also with the fact that both *Mundinia* and *Sauroleishmania* had switched to the new hosts or vectors. The majority of dixenous Leishmaniinae (i.e. *Leishmania*, *Paraleishmania* and *Endotrypanum*) parasitize mammals and are transmitted by phlebotomine sand flies and this, therefore, is the most likely ancestral variant of the life cycle. Meanwhile, *Sauroleishmania* spp. switched their vertebrate host from mammals to reptiles, whereas *Mundinia* spp. have substituted the phlebotomine sand fly hosts with biting midges and/or non-conventional sand flies. We speculate that adaptation to the new hosts or vectors has led to different, possibly simplified, host-parasite relationships and, thereby, made some of the previously used proteins redundant. Indeed, *Sauroleishmania* spp*.* demonstrate less specific relationships with their vertebrate hosts as compared to other *Leishmania* spp. Their promastigotes usually reside in the intestine or in the bloodstream, while occasionally formed amastigotes do not survive in macrophages [106].

Little is known about the relationships of *L.* (*Mundinia*) spp. and their vectors. However, our finding of a significant shrinkage of repertoires of the SCGs and SCAs in *Mundinia*, which are involved in interactions of promastigotes with the insect gut, implies simplification of the host-parasite relationships. At the same time, amastins and PIG-Y, which are primarily important for the survival of amastigotes in macrophages, showed generally the same evolutionary trends as in *L.* (*Leishmania*) and *L.* (*Viannia*), i.e. underwent independent losses. Moreover, those were mainly β-amastins, which are expressed in the vectorial part of the life cycle in *T. cruzi* [79]. In contrast, *Sauroleishmania* lost all amastigote-specific δ-amastins [41], whereas all other *Leishmania* subgenera preserved them.

In summary, we propose that the evolution of genomes in the genus *Leishmania* and, in particular, in the subgenus *Mundinia* was mainly shaped by host (or vector) switches.

## Conclusions

In this work we have sequenced and analyzed genomes of several representatives of the most understudied *Leishmania* subgenus, *Mundinia*. Comparative analyses allowed us to gain additional insights into the origin of pathogenic *Leishmania*. We propose that the evolution of this genus was mainly driven by the host (or vector) switches.

## Supplementary information

**Additional file 1: Figure S1.** BlobTools statistics for *L*. (*M*.) *enriettii* MCAV/BR/1945/LV90 before filtering.

**Additional file 2: Figure S2.** BlobTools statistics for *L*. (*M*.) *enriettii* MCAV/BR/1945/LV90 after filtering.

**Additional file 3: Figure S3.** BlobTools statistics for *L*. (*M*.) *macropodum* MMAC/AU/2004/AM-2004 before filtering.

**Additional file 4: Figure S4.** BlobTools statistics for *L*. (*M*.) *macropodum* MMAC/AU/2004/AM-2004 after filtering.

**Additional file 5: Figure S5.** BlobTools statistics for *L*. (*M*.) *martiniquensis* MHOM/MQ/1992/MAR1 before filtering.

**Additional file 6: Figure S6.** BlobTools statistics for *L*. (*M*.) *martiniquensis* MHOM/MQ/1992/MAR1 after filtering.

**Additional file 7: Figure S7.** CovPlot statistics for the final assembly of *L*. (*M*.) *enriettii* MCAV/BR/1945/LV90.

**Additional file 8: Figure S8.** CovPlot statistics for the final assembly of *L*. (*M*.) *macropodum* MMAC/AU/2004/AM-2004.

**Additional file 9: Figure S9.** CovPlot statistics for the final assembly of *L*. (*M*.) *martiniquensis* MHOM/MQ/1992/MAR1.

**Additional file 10: Figure S10.** Plot showing the distribution of genomic read coverage values for the genome assemblies of *L*. (*M*.) *enriettii* (blue line), *L*. (*M*.) *martiniquensis* (orange), *L*. (*M*.) *macropodum* (green).

**Additional file 11: Figure S11.** Panel (A). Schematic representation of the two-way synteny between the genomes of *Leishmania* (*Mundinia*) strains sequenced in this study and the ones available in TriTrypDB, as well as between *Leishmania* (*Mundinia*) and *L. major* Friedlin. Corresponding syntenic blocks are connected with red ribbons. In each case scaffolds of two compared strains/species are filled with different colors and are separated by a blank space. Only the chromosomes which actually have synteny blocks are shown. Panel (B). Summary statistics for pairwise synteny analyses among *Leishmania* (*Mundinia*) strains and the reference genome sequence of *L. major* Friedlin.

**Additional file 12: Figure S12.** Maximum-Likelihood phylogenetic tree of trypanosomatid amastins. The tree was inferred using IQ-TREE v.1.5.3 with the JTT + I + G4 model and 1000 bootstrap replicates. The support values are in the following format: SH-aLRT support (%)/bootstrap support (%).

**Additional file 13: Figure S13.** Maximum-Likelihood phylogenetic tree of trypanosomatid phosphatydylinositol glycan class Y (PIG-Y) sequences. The tree was inferred using IQ-TREE v.1.5.3 with the JTT + I + G4 model and 1000 bootstrap replicates. The support values are in the following format: SH-aLRT support (%)/bootstrap support (%).

**Additional file 14: Figure S14.** Maximum-Likelihood phylogenetic tree of trypanosomatid side chain galactosyltransferases (SCGs) and side chain arabinosyltransferases (SCAs) sequences. The tree was inferred using IQ-TREE v.1.5.3 with 1000 bootstrap replicates and VT + F + I + G4 and JTT + F + G4 models for SCGs and SCAs, respectively. The support values are in the following format: SH-aLRT support (%)/bootstrap support (%). Reference SCGs and SCAs of *L. major* are highlighted in color.

**Additional file 15: Figure S15.** Maximum-Likelihood phylogenetic tree of ferrochelatase sequences. The tree was constructed using IQ-TREE v.1.5.3 with 1000 bootstrap replicates and JTT + I + G4 model. The support values are in the following format: SH-aLRT support (%)/bootstrap support (%).

**Additional file 16: Table S1.** Dataset used in this study. Organisms, whose genomes and transcriptomes were sequenced in this work, are in bold.

**Additional file 17: Table S2.** Estimates of evolutionary divergence among putative amastin sequences. The number of amino acid differences per site from between sequences are shown. The analysis involved 450 amino acid sequences. All ambiguous positions were removed for each sequence pair. There were a total of 1532 positions in the final dataset. Evolutionary analyses were conducted in MEGA7.

**Additional file 18: Table S3.** Estimates of evolutionary divergence among putative side chain galactosyltransferase sequences. The number of amino acid differences per site from between sequences are shown. The analysis involved 87 amino acid sequences. All positions with less than 80% site coverage were eliminated. Less than 20% alignment gaps, missing data and ambiguous bases were allowed at any position. There were a total of 476 positions in the final dataset. Evolutionary analyses were conducted in MEGA7. The presence of n/c in the results denotes cases in which it was not possible to estimate evolutionary distances.

**Additional file 19: Table S4.** Estimates of evolutionary divergence among putative side chain arabinosyltransferase sequences. The number of amino acid differences per site from between sequences are shown. The analysis involved 20 amino acid sequences. All positions with less than 80% site coverage were eliminated. Less than 20% alignment gaps, missing data and ambiguous bases were allowed at any position. There were total of 694 positions in the final dataset. Evolutionary analyses were conducted in MEGA7.

**Additional file 20: Table S5.** Estimates of evolutionary divergence among putative phosphatydylinositol glycan class Y (PIG-Y) sequences. The number of amino acid differences per site from between sequences are shown. The analysis involved 14 amino acid sequences. All ambiguous positions were removed for each sequence pair. There were a total of 128 positions in the final dataset. Evolutionary analyses were conducted in MEGA7.

**Additional file 21: Table S6.** Estimates of evolutionary divergence among putative ferrochelatase sequences. The number of amino acid differences per site from between sequences are shown. The analysis involved 45 amino acid sequences. All positions with less than 80% site coverage were eliminated. Less than 20% alignment gaps, missing data and ambiguous bases were allowed at any position. There were a total of 247 positions in the final dataset. Evolutionary analyses were conducted in MEGA7. The presence of n/c in the results denotes cases in which it was not possible to estimate evolutionary distances.

**Additional file 22: Table S7.** Summary statistics for *Mundinia* genomes sequenced in this study and several publicly available trypanosomatid genome assemblies.

**Additional file 23: Table S8.** Ploidy estimates for fifty longest scaffolds and all pseudochromosome level sequences of *Leishmania* (*Mundinia*) spp. sequenced in this study.

**Additional file 24: Table S9.** Gene gains and losses at the *Leishmania* (*Mundinia*) node.

**Additional file 25: Table S10.** Gene gains and losses at the *Leishmania*/ *Sauroleishmania*/ *Viannia* node.

**Additional file 26: Table S11.** Gene family expansion and contractions at the *Leishmania* (*Mundinia*) node.

**Additional file 27: Table S12.** Gene family expansion and contractions at the *Leishmania*/ *Sauroleishmania*/ *Viannia* node.

## Data Availability

The datasets generated and analyzed during the current study will be available in the NCBI SRA repository under accession numbers SRX5006814, SRX5006815, and SRX5006816 (Bioproject: PRJNA505413) upon publication, https://www.ncbi.nlm.nih.gov/sra

## Abbreviations

GPI: Glycosylphosphatidylinositol
OG: Orthogroup
PIG-Y: Phosphatydylinositol glycan class Y protein
SCA: Side chain arabinosyltransferase
SCG: Side chain galactosyltransferases
