# Supplementary figures and images for: Comparative genomics of Leishmania (Mundinia)
Source: BMC Genomics. 2019 Oct 11;20:726. doi: 10.1186/s12864-019-6126-y (PMC6787982; doi:10.1186/s12864-019-6126-y)

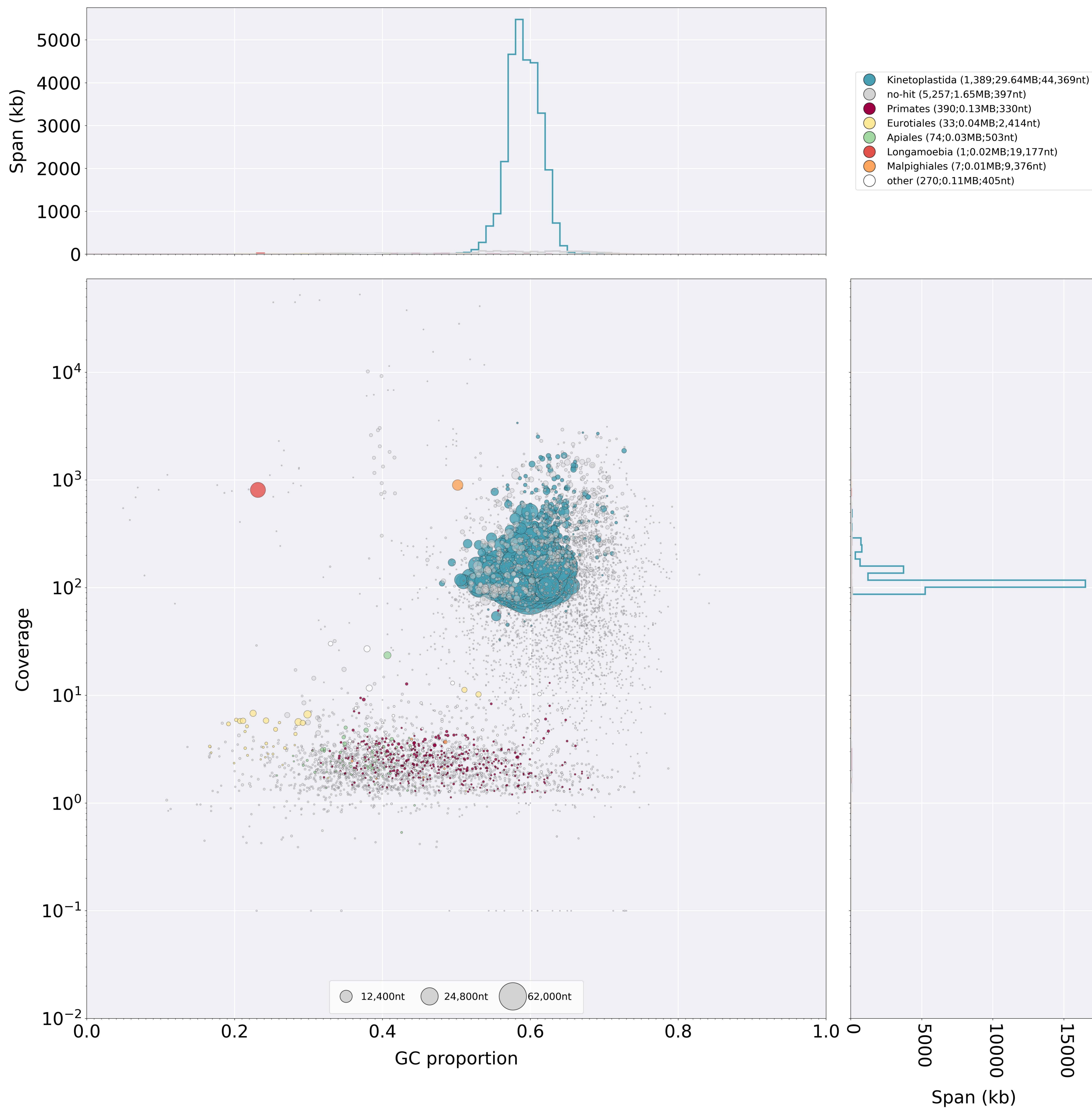

Supplement: Supplementary file 1 — Additional file 1: Figure S1. BlobTools statistics for L. (M.) enriettii MCAV/BR/1945/LV90 before filtering. [file 12864_2019_6126_MOESM1_ESM.pdf]

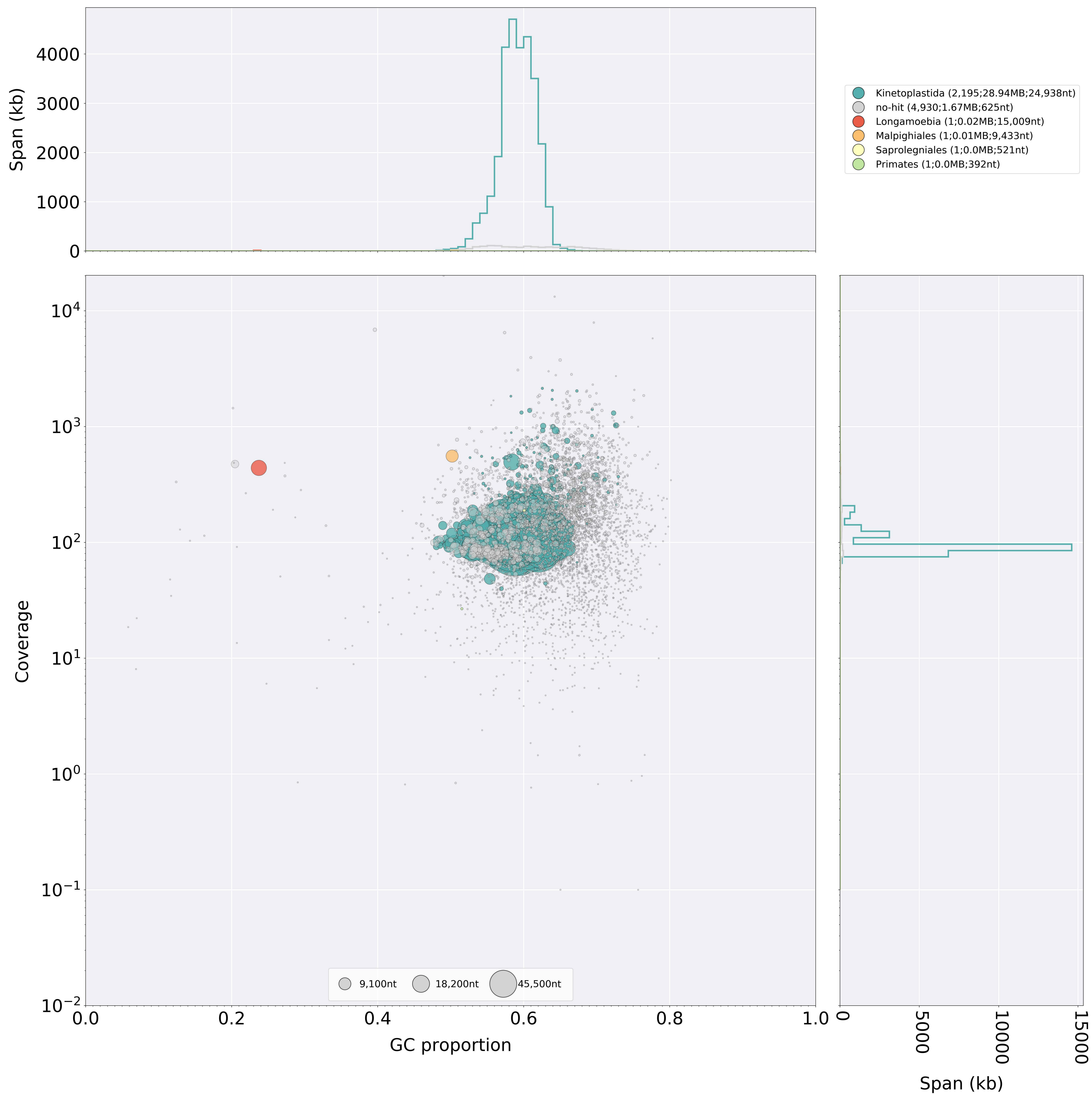

Supplement: Supplementary file 2 — Additional file 2: Figure S2. BlobTools statistics for L. (M.) enriettii MCAV/BR/1945/LV90 after filtering. [file 12864_2019_6126_MOESM2_ESM.pdf]

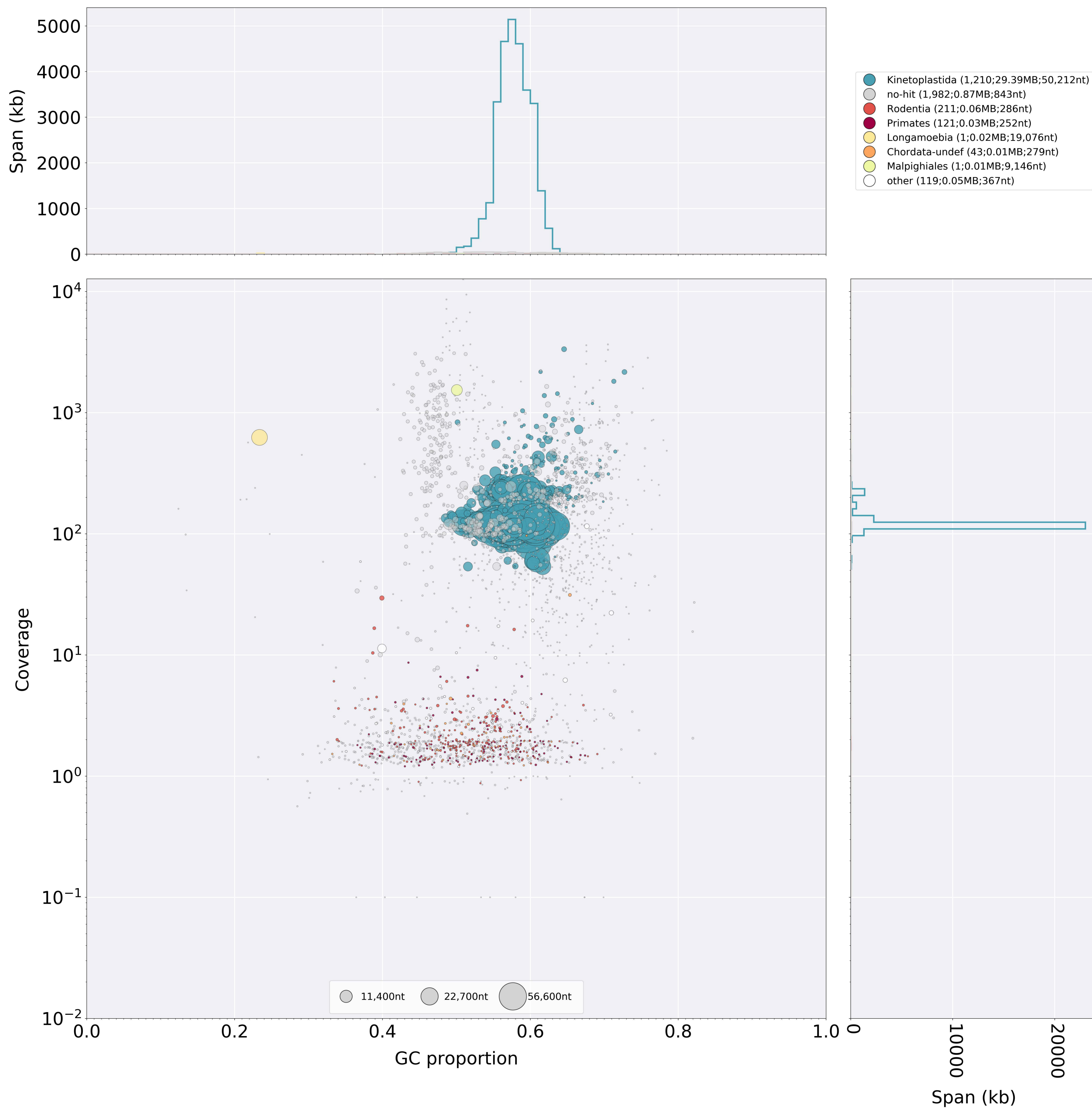

Supplement: Supplementary file 3 — Additional file 3: Figure S3. BlobTools statistics for L. (M.) macropodum MMAC/AU/2004/AM-2004 before filtering. [file 12864_2019_6126_MOESM3_ESM.pdf]

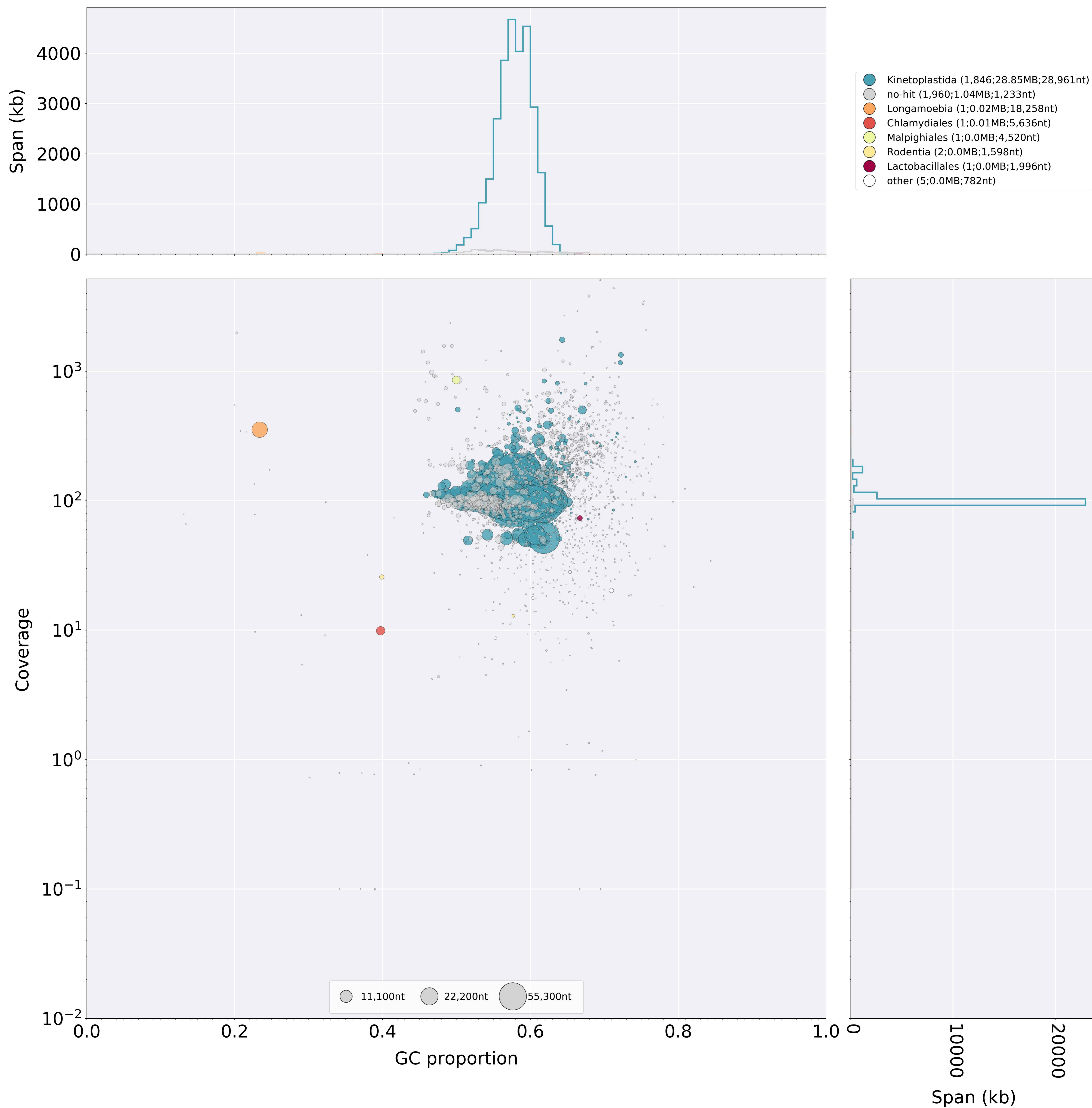

Supplement: Supplementary file 4 — Additional file 4: Figure S4. BlobTools statistics for L. (M.) macropodum MMAC/AU/2004/AM-2004 after filtering. [file 12864_2019_6126_MOESM4_ESM.pdf]

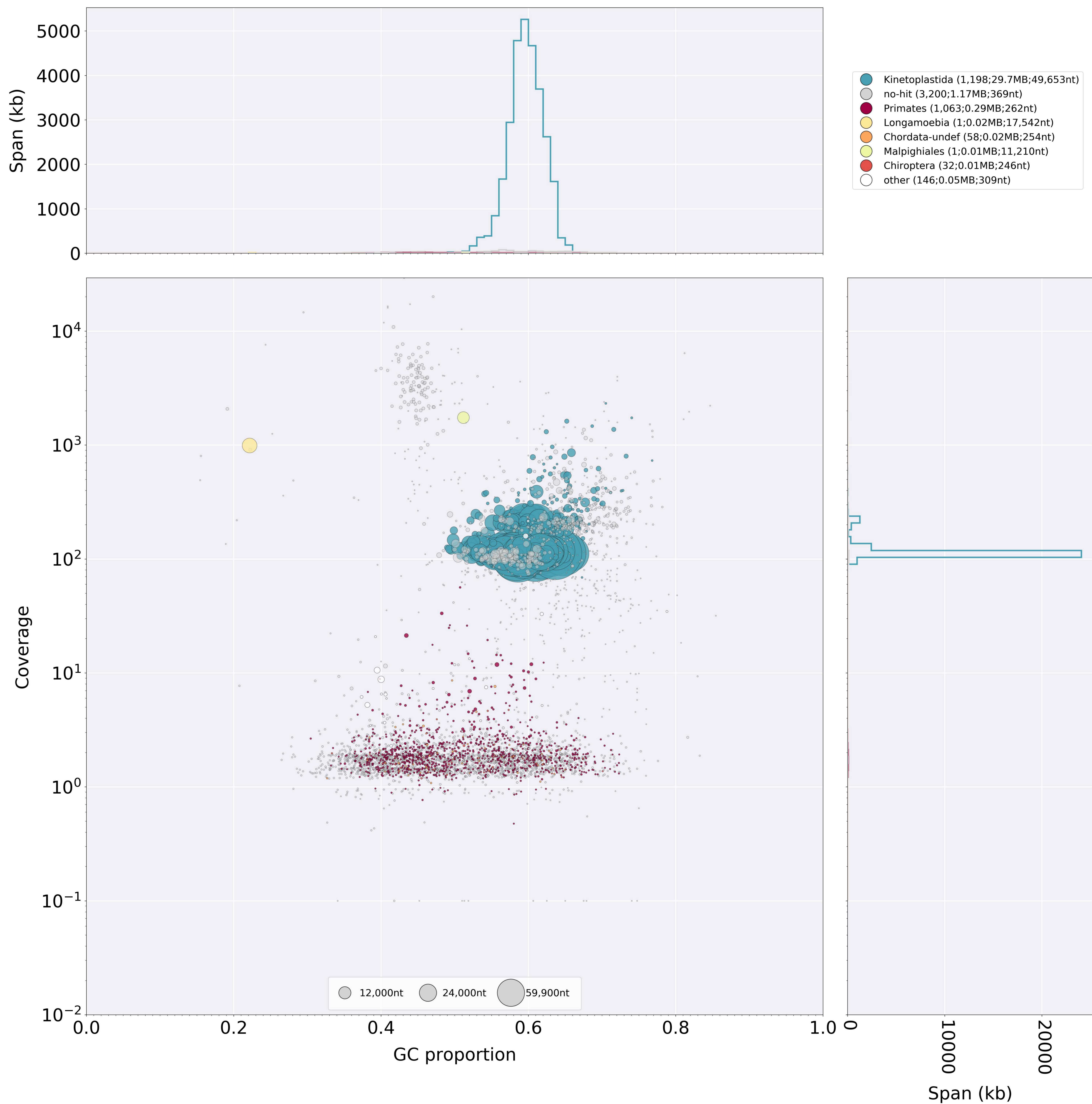

Supplement: Supplementary file 5 — Additional file 5: Figure S5. BlobTools statistics for L. (M.) martiniquensis MHOM/MQ/1992/MAR1 before filtering. [file 12864_2019_6126_MOESM5_ESM.pdf]

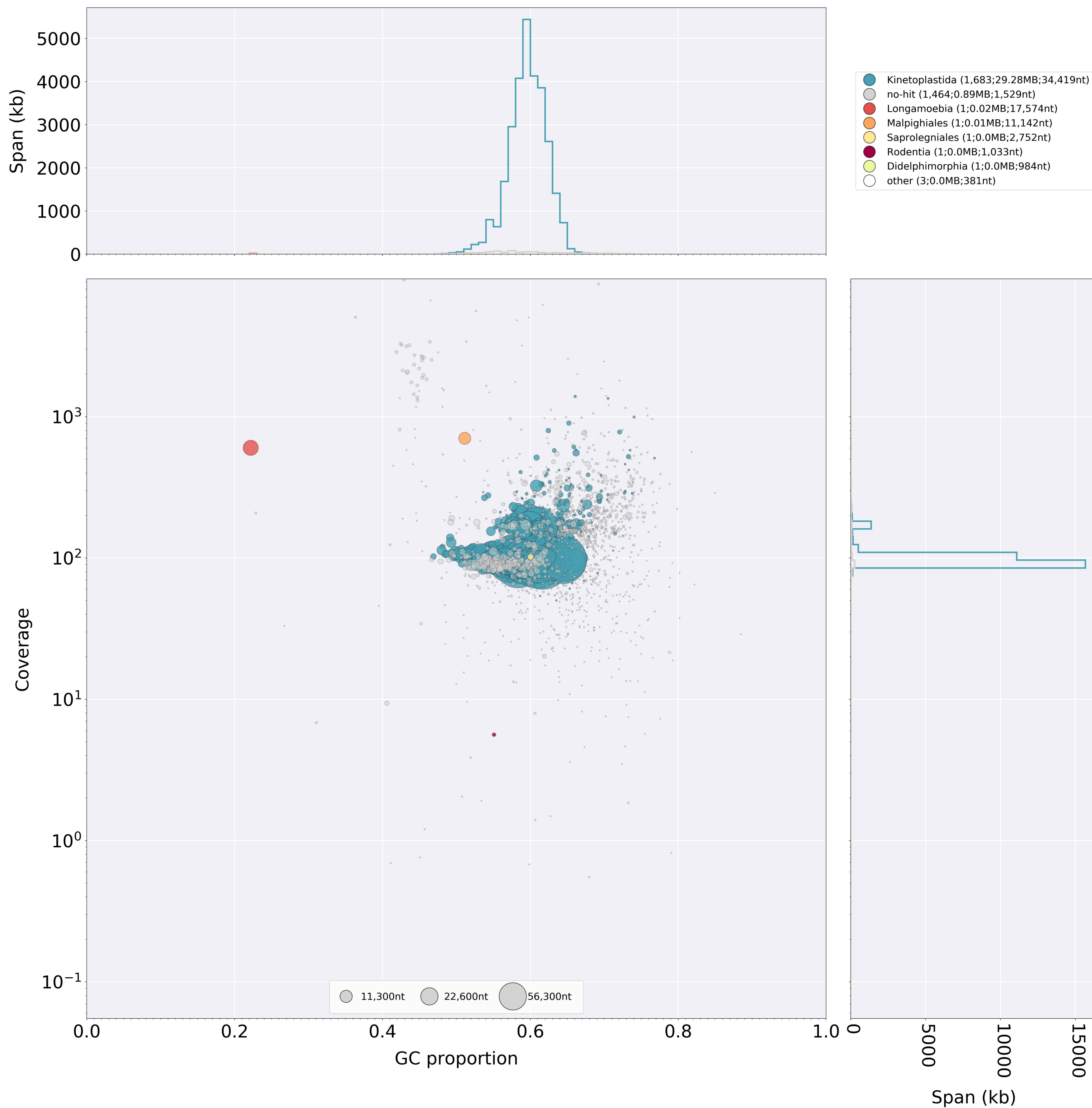

Supplement: Supplementary file 6 — Additional file 6: Figure S6. BlobTools statistics for L. (M.) martiniquensis MHOM/MQ/1992/MAR1 after filtering. [file 12864_2019_6126_MOESM6_ESM.pdf]

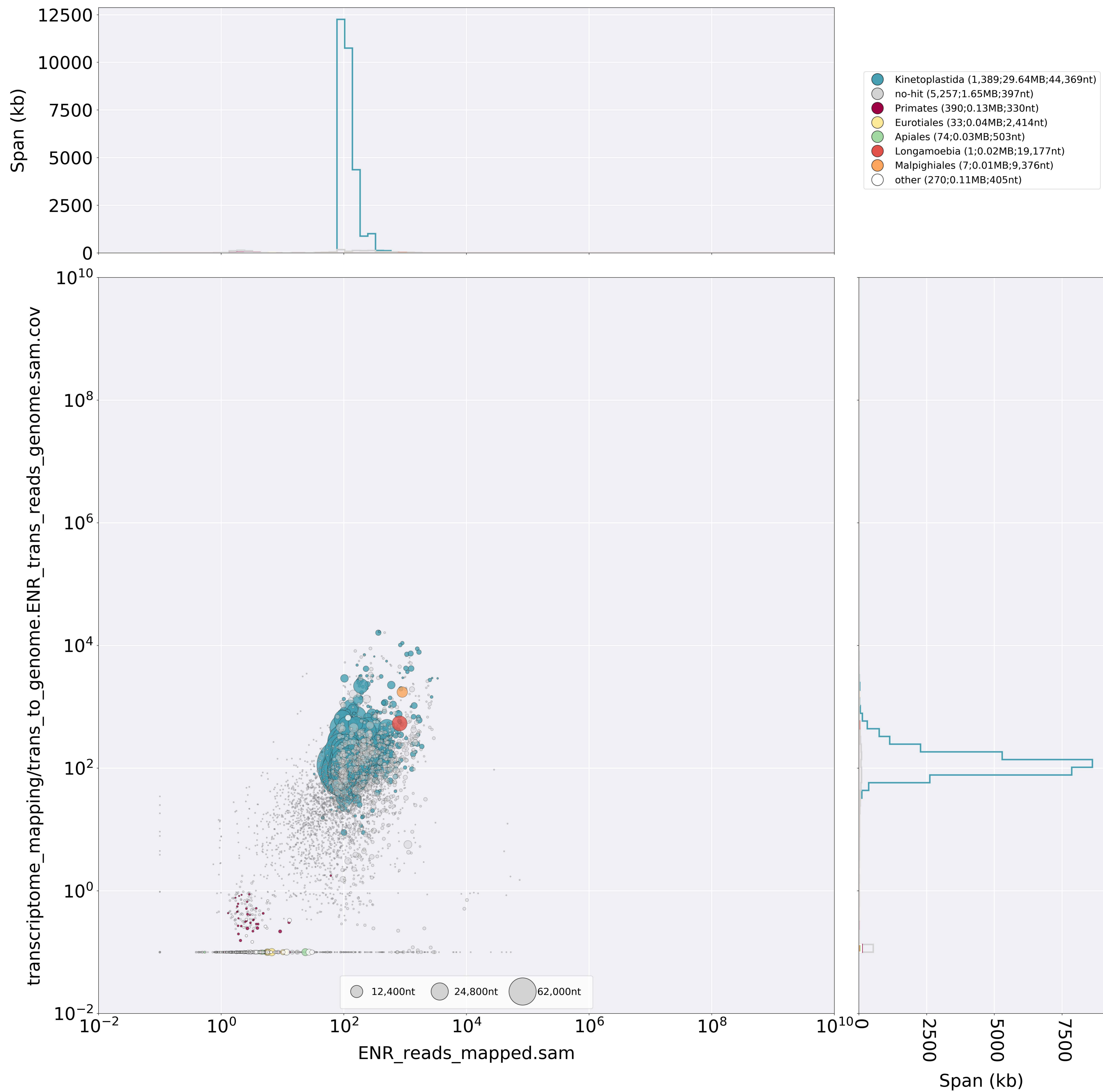

Supplement: Supplementary file 7 — Additional file 7: Figure S7. CovPlot statistics for the final assembly of L. (M.) enriettii MCAV/BR/1945/LV90. [file 12864_2019_6126_MOESM7_ESM.pdf]

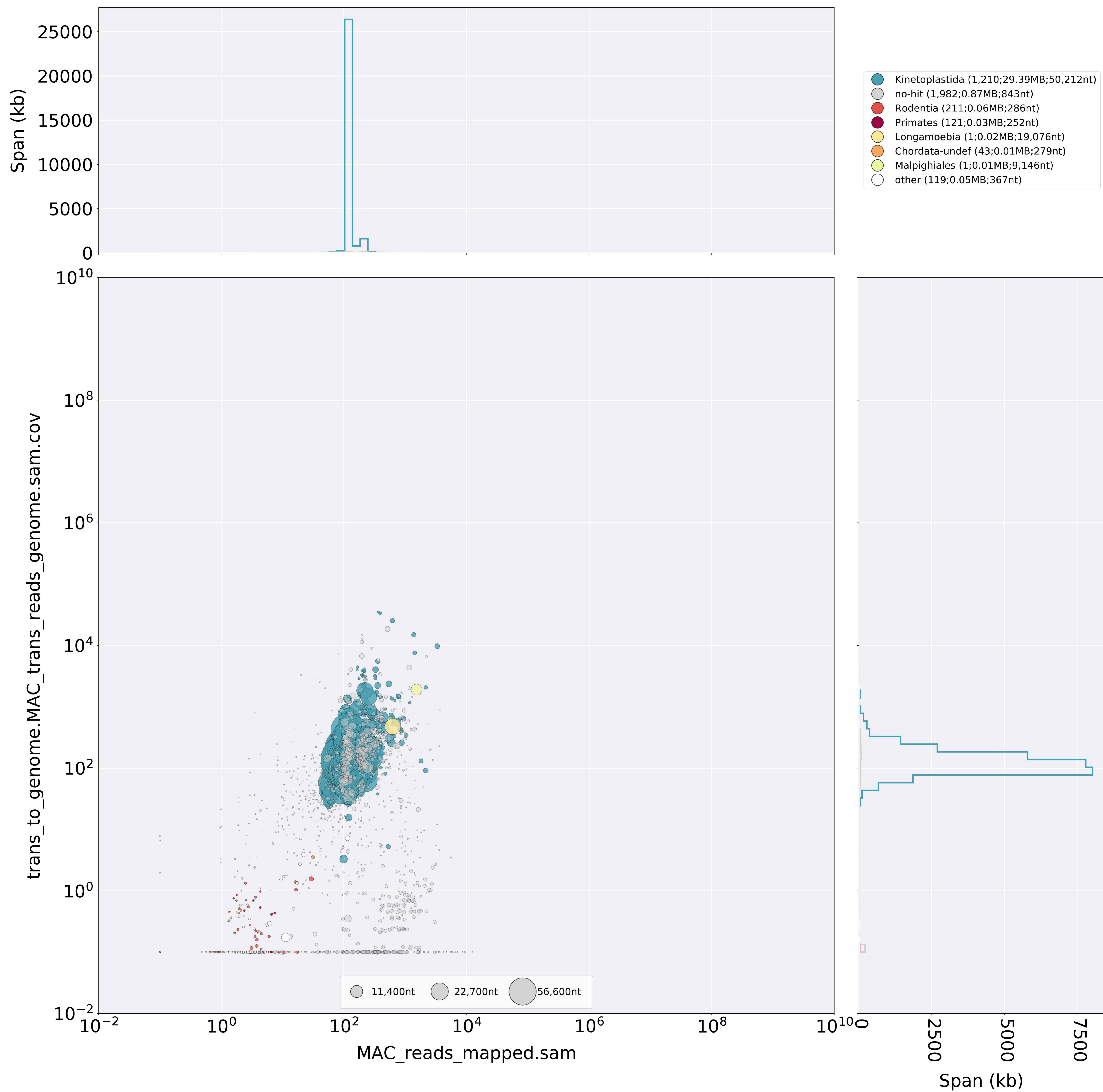

Supplement: Supplementary file 8 — Additional file 8: Figure S8. CovPlot statistics for the final assembly of L. (M.) macropodum MMAC/AU/2004/AM-2004. [file 12864_2019_6126_MOESM8_ESM.pdf]

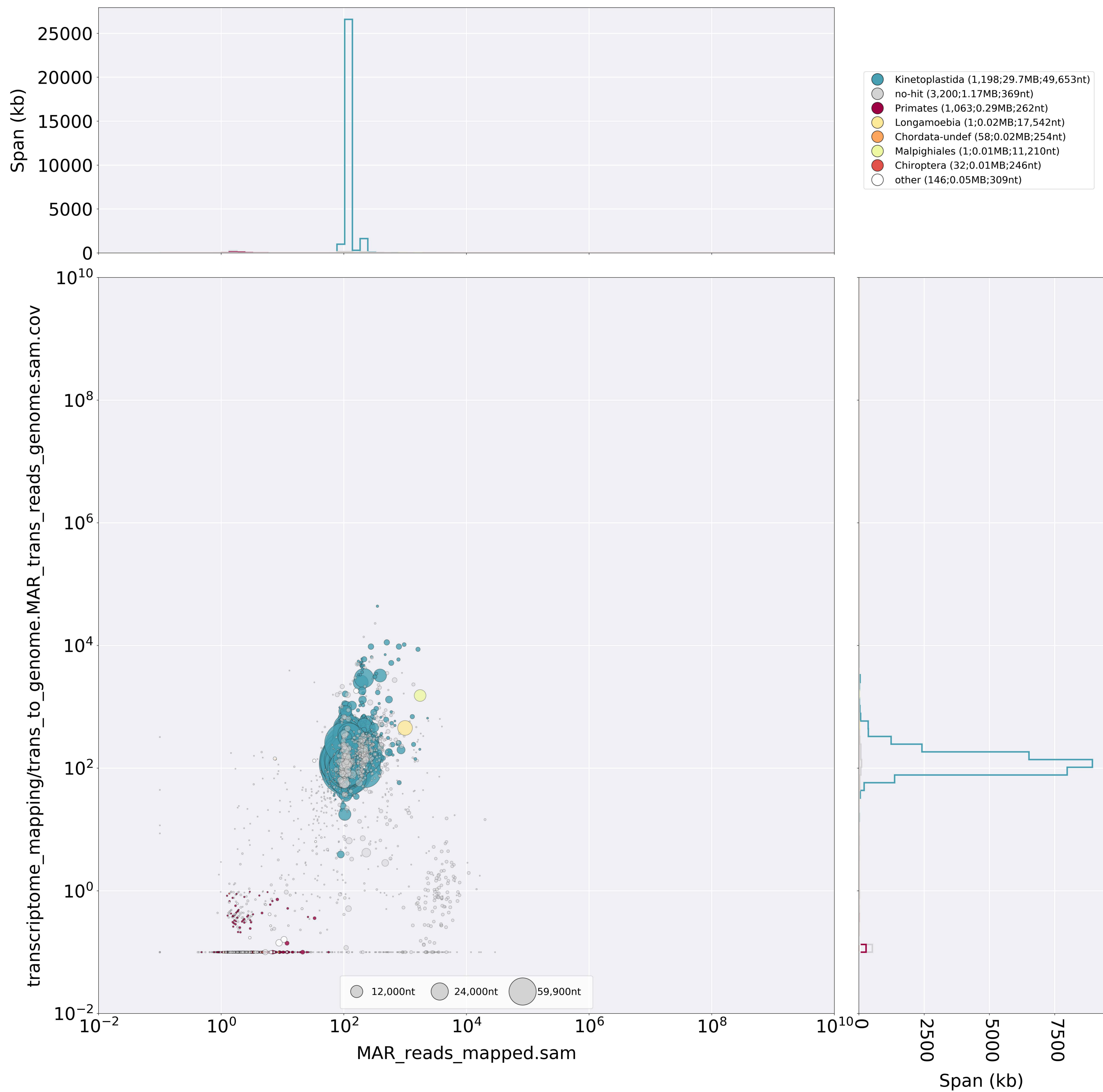

Supplement: Supplementary file 9 — Additional file 9: Figure S9. CovPlot statistics for the final assembly of L. (M.) martiniquensis MHOM/MQ/1992/MAR1. [file 12864_2019_6126_MOESM9_ESM.pdf]

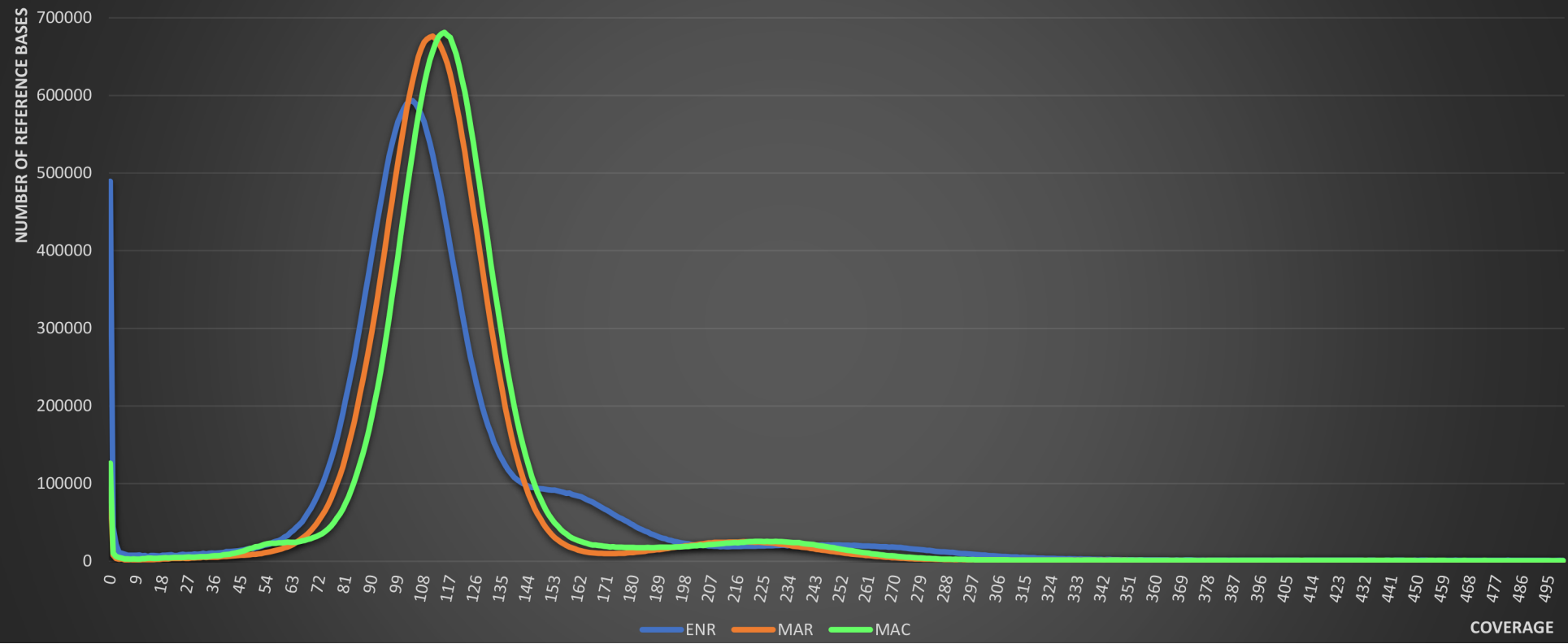

Supplement: Supplementary file 10 — Additional file 10: Figure S10. Plot showing the distribution of genomic read coverage values for the genome assemblies of L. (M.) enriettii (blue line), L. (M.) martiniquensis (orange), L. (M.) macropodum (green). [file 12864_2019_6126_MOESM10_ESM.pdf]

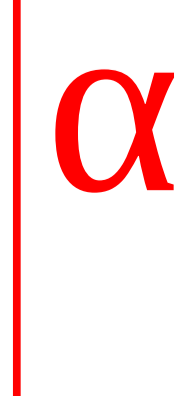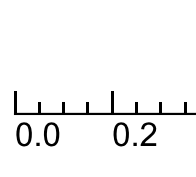

Supplement: Supplementary file 12 — Additional file 12: Figure S12. Maximum-Likelihood phylogenetic tree of trypanosomatid amastins. The tree was inferred using IQ-TREE v.1.5.3 with the JTT + I + G4 model and 1000 bootstrap replicates. The support values are in the following format: SH-aLRT support (%)/bootstrap support (%). [file 12864_2019_6126_MOESM12_ESM.pdf]

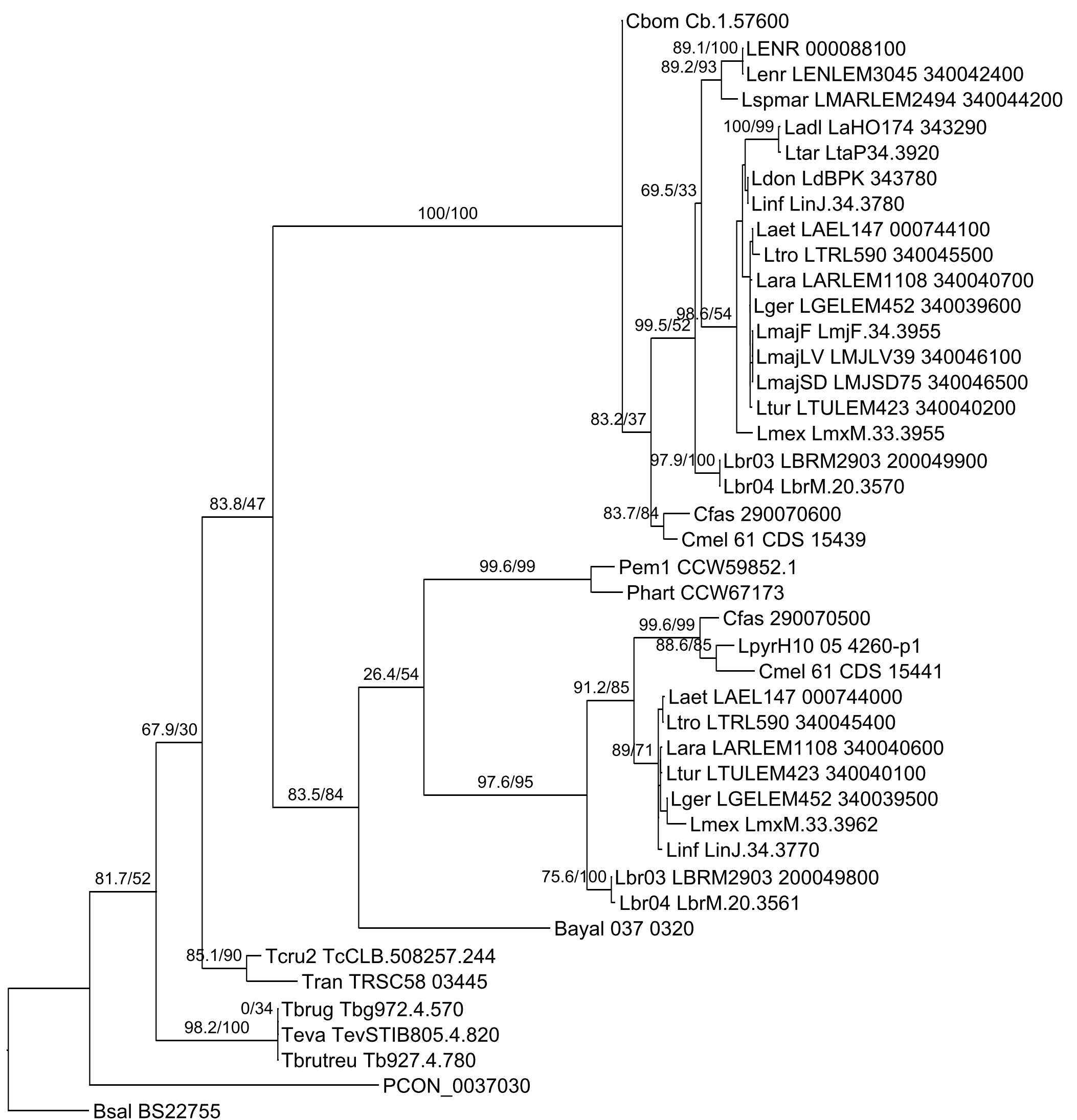

0.0 0.2

Supplement: Supplementary file 13 — Additional file 13: Figure S13. Maximum-Likelihood phylogenetic tree of trypanosomatid phosphatydylinositol glycan class Y (PIG-Y) sequences. The tree was inferred using IQ-TREE v.1.5.3 with the JTT + I + G4 model and 1000 bootstrap replicates. The support values are in the following format: SH-aLRT support (%)/bootstrap support (%). [file 12864_2019_6126_MOESM13_ESM.pdf]

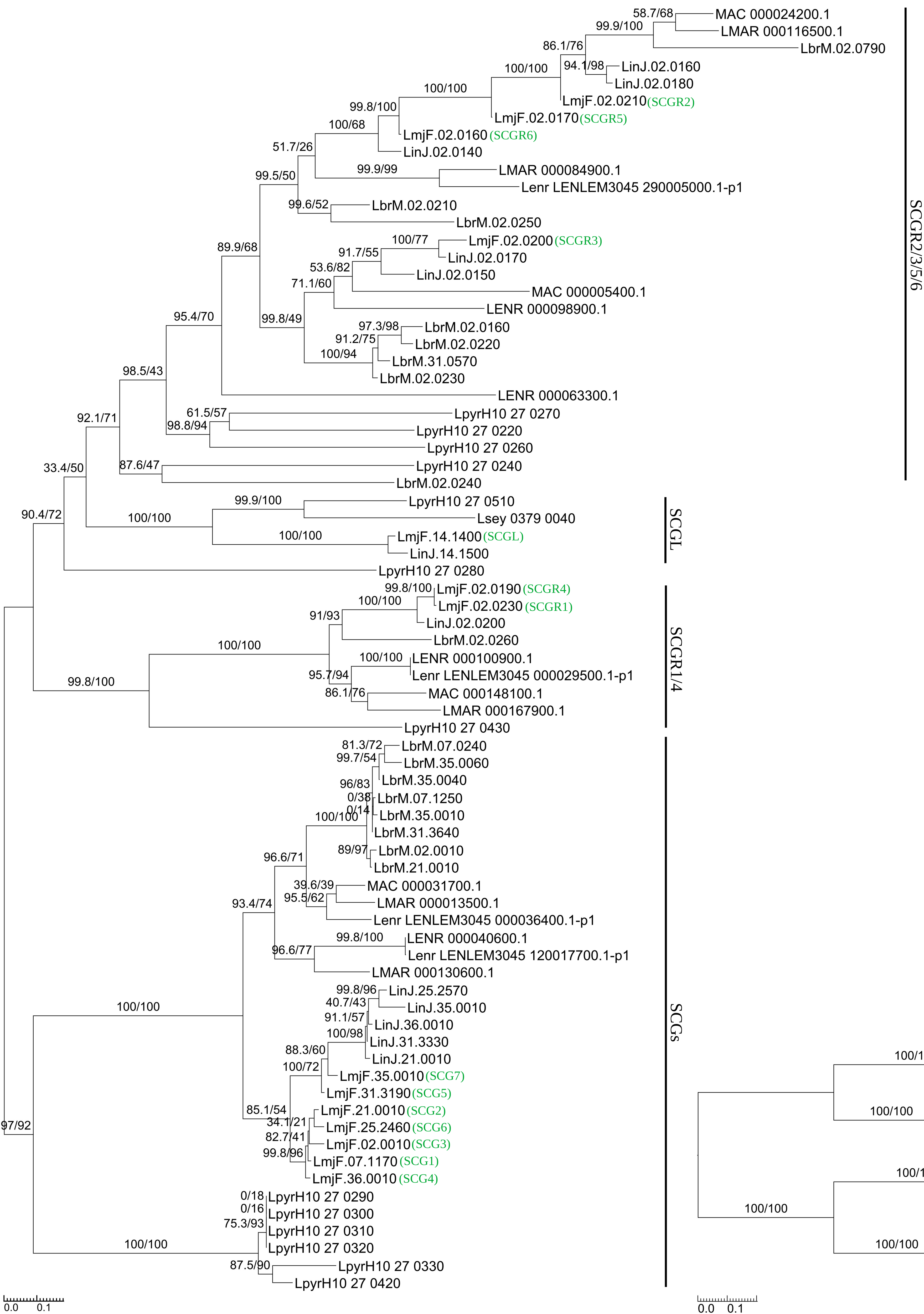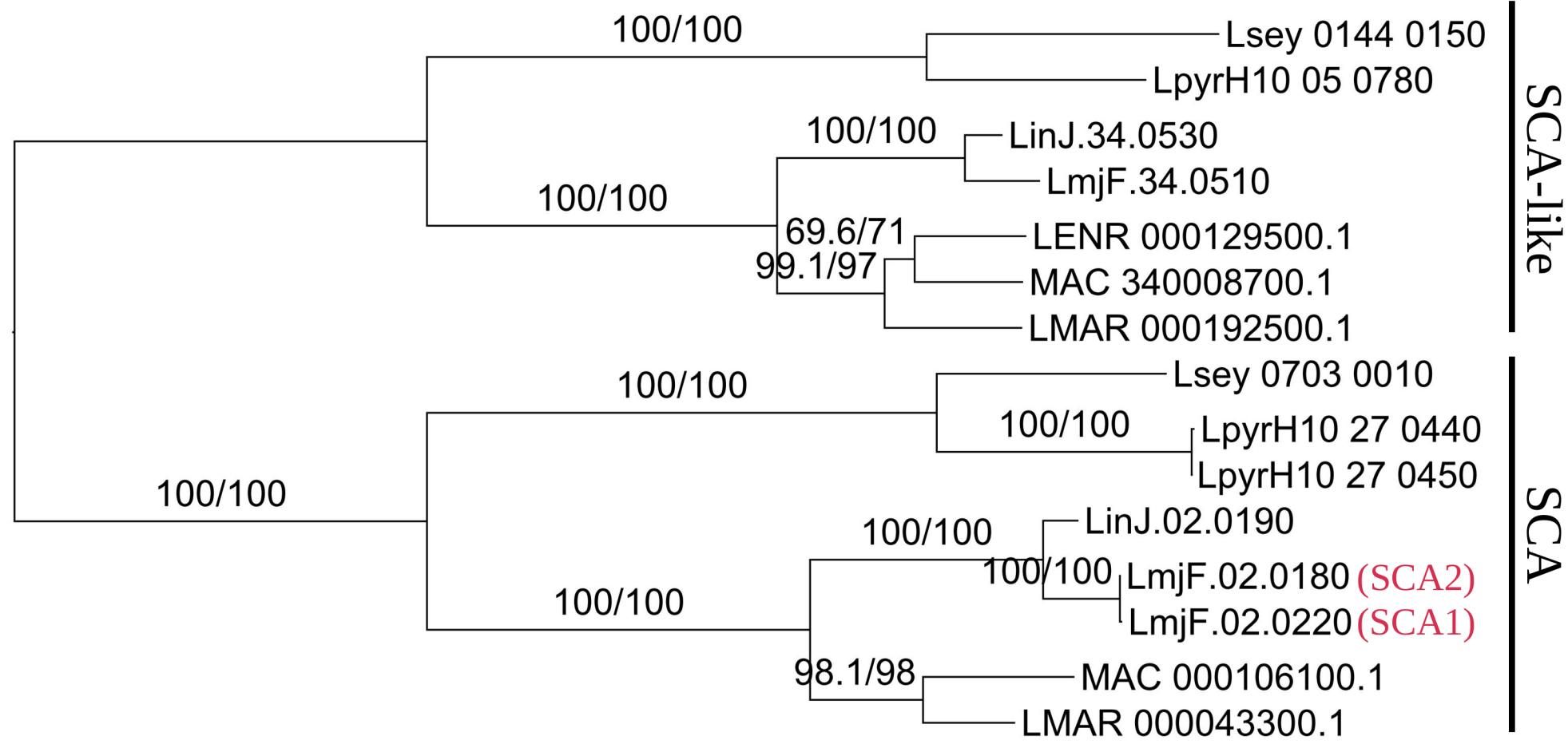

Supplement: Supplementary file 14 — Additional file 14: Figure S14. Maximum-Likelihood phylogenetic tree of trypanosomatid side chain galactosyltransferases (SCGs) and side chain arabinosyltransferases (SCAs) sequences. The tree was inferred using IQ-TREE v.1.5.3 with 1000 bootstrap replicates and VT + F + I + G4 and JTT + F + G4 models for SCGs and SCAs, respectively. The support values are in the following format: SH-aLRT support (%)/bootstrap support (%). Reference SCGs and SCAs of L. major are highlighted in color. [file 12864_2019_6126_MOESM14_ESM.pdf]

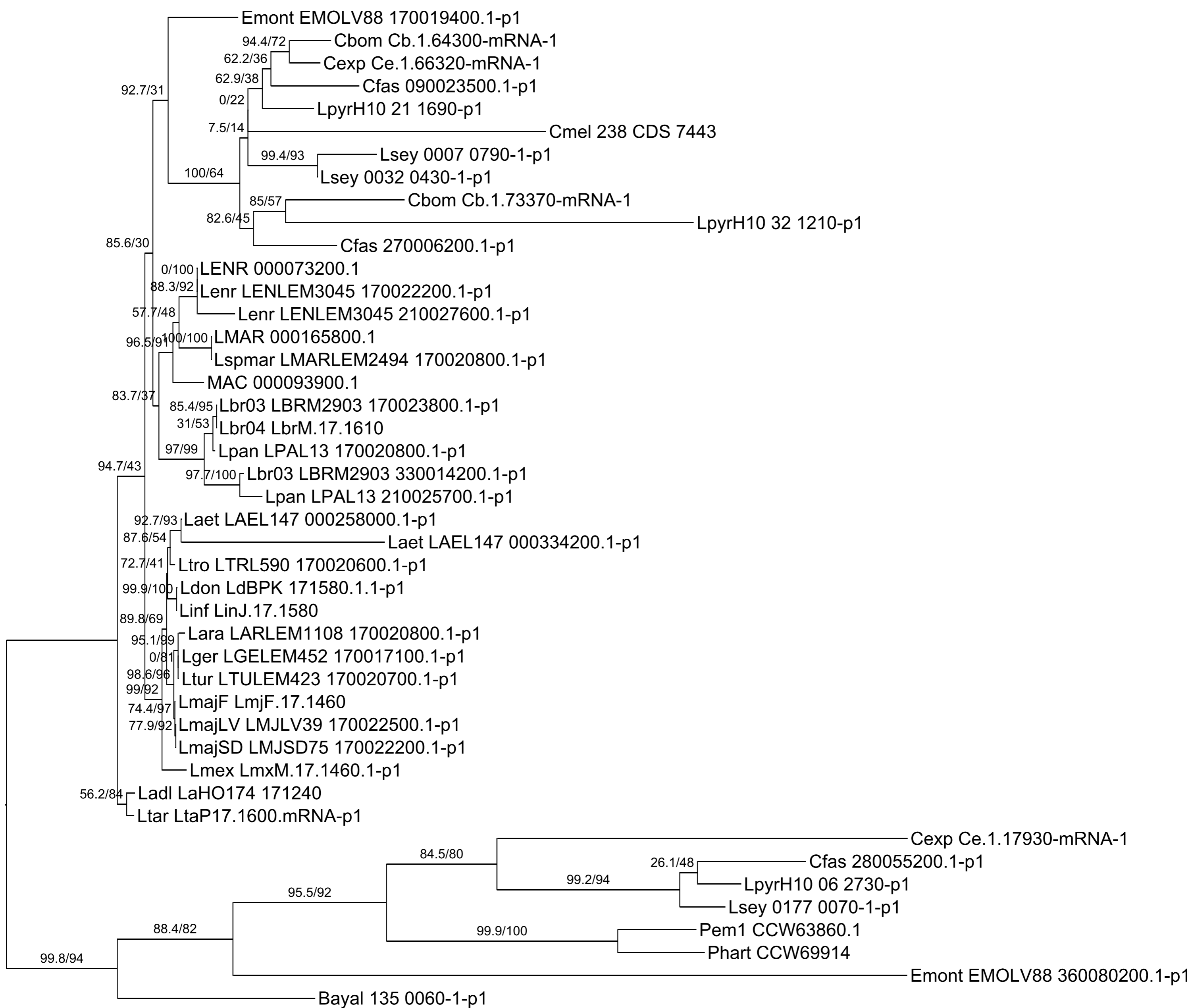

0.0 0.1

Supplement: Supplementary file 15 — Additional file 15: Figure S15. Maximum-Likelihood phylogenetic tree of ferrochelatase sequences. The tree was constructed using IQ-TREE v.1.5.3 with 1000 bootstrap replicates and JTT + I + G4 model. The support values are in the following format: SH-aLRT support (%)/bootstrap support (%). [file 12864_2019_6126_MOESM15_ESM.pdf]
