# Supplementary material for: Comparative genomics of Leishmania (Mundinia)
Source: BMC Genomics. 2019 Oct 11;20:726. doi: 10.1186/s12864-019-6126-y (PMC6787982; doi:10.1186/s12864-019-6126-y)
Supplement: Supplementary file 11 — Additional file 11: Figure S11. Panel (A). Schematic representation of the two-way synteny between the genomes of Leishmania (Mundinia) strains sequenced in this study and the ones available in TriTrypDB, as well as between Leishmania (Mundinia) and L. major Friedlin. Corresponding syntenic blocks are connected with red ribbons. In each case scaffolds of two compared strains/species are filled with different colors and are separated by a blank space. Only the chromosomes which actually have synteny blocks are shown. Panel (B). Summary statistics for pairwise synteny analyses among Leishmania (Mundinia) strains and the reference genome sequence of L. major Friedlin. [file 12864_2019_6126_MOESM11_ESM.pdf]

A

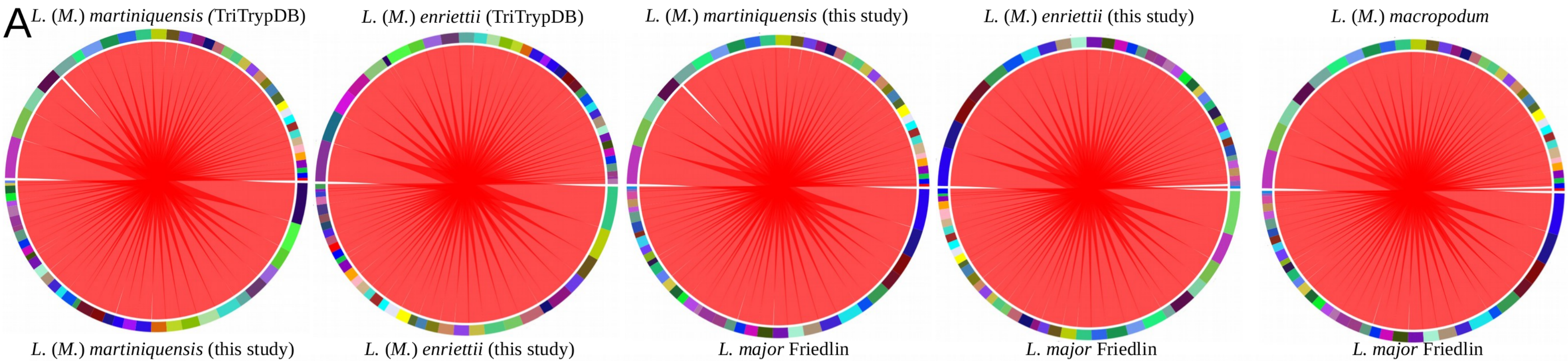

B

Species	# genes	% of sequence covered by genes	# of anchors	% of anchors in blocks	% of anchors annotated	% of coverage by anchors	# of blocks	% double coverage	# of inverted blocks	% of genes within synteny blocks
Leishmania martiniquensis MHOM/MQ/1992/MAR1 (this study)	30,427	41	4,105	62	78	97	37	0	1	98
Leishmania martiniquensis MHOM/MQ/1992/MAR1 (TriTrypDB)	30,814	49	4,105	62	87	96	37	0	1	80
Leishmania enriettii MCAV/BR/1945/LV90 (this study)	30,285	42	6,083	62	67	94	36	0	0	98
Leishmania enriettii MCAV/BR/1995/CUR3 (TriTrypDB)	30,761	49	6,083	62	76	93	36	0	0	78
Leishmania martiniquensis MHOM/MQ/1992/MAR1 (this study)	30,427	41	8,144	75	82	52	44	0	7	93
Leishmania major Friedlin	32,855	50	8,144	75	97	50	44	0	7	66
Leishmania enriettii MCAV/BR/1945/LV90 (this study)	30,285	42	8,039	74	81	54	40	0	2	94
Leishmania major Friedlin	32,855	50	8,039	74	95	49	40	0	2	93
Leishmania macropodum MMAC/AU/2004/AM-2004	30,120	42	8,316	74	96	54	40	0	2	94
Leishmania major Friedlin	32,855	50	8,316	74	82	51	40	0	2	66
